# Coherent Signal DOA Estimation and 3D Point Cloud Imaging Based on a Straight-Curved Hybrid L-Shaped Conformal Array

**DOI:** 10.3390/s26175445

**Published:** 2026-08-28

**Authors:** Bowen Bie, Yang Chen, Huiwen Chen, Ning Li

**Affiliations:** 1 School of Information Mechanics and Sensing Engineering, Xidian University, Xi’an 710071, China; biebowen@gmail.com (B.B.); 25261215960@stu.xidian.edu.cn (Y.C.); 2 Hangzhou Institute of Technology, Xidian University, Xi’an 710071, China; 23021212012@stu.xidian.edu.cn; 3 Faculty of Infor-X, School of Information Mechanics and Sensing Engineering, Xidian University, Xi’an 710071, China

**Keywords:** millimeter-wave radar, L-shaped conformal array, virtual array transformation, 2D DOA estimation, 3D point cloud imaging

## Abstract

**Highlights:**

**What are the main findings?**

- A straight-curved hybrid L-shaped conformal array topology tailored for cylindrical radomes is proposed, with an orthogonal virtual manifold transformation that compensates for non-linear phase distortion induced by the conformal surface.
- A cascaded Forward-Backward Spatial Smoothing (FBSS) and Root-MUSIC framework is developed for efficient coherent signal decoherence, with a Frobenius-norm-based global cost function that pairs independent angles and suppresses spatial ghost targets.

**What are the implications of the main findings?**

- Systematic 3D computer vision evaluations demonstrate a median point-wise localization error of 0.3986 m, CD of 0.9695 m^2^, EMD of 1.9504 m, and an angular resolution of 1.0° under the stated simulation conditions; the algorithm maintains stable 3D reconstruction around a post-pulse-compression SNR of −8.0 dB and a simulation-based curvature threshold of 12.50 m^−1^ (r=0.08 m).
- This framework provides a data processing pipeline for high-resolution conformal radar 3D perception and offers theoretical support and dimensional design references for next-generation airborne radar system design.

**Abstract:**

High-speed airborne platforms impose stringent aerodynamic constraints that restrict traditional planar antenna designs. Concurrently, multi-component echoes from extended targets and complex propagation environments induce strong signal coherence, severely degrading spatial angle estimation. To address these dual challenges, this paper proposes a straight-curved hybrid L-shaped asymmetric conformal array hardware topology tailored for cylindrical radomes. Building upon this, a millimeter-wave radar 3D point cloud imaging framework is developed for coherent targets. An orthogonal virtual manifold transformation is first devised to effectively compensate for the non-linear phase distortion induced by the conformal topology. Subsequently, a cascaded Forward-Backward Spatial Smoothing (FBSS) and Root-MUSIC framework is used for efficient signal decoherence. To resolve angle mismatches, a global cost function based on the cross-covariance Frobenius norm is formulated, which pairs independent angles and significantly suppresses spatial ghost targets. Systematic evaluations using 3D computer vision metrics demonstrate that the proposed method achieves accurate geometric restoration of aircraft targets with coherent signals. In the representative simulation, the method obtains a median point-wise localization error of 0.3986 m, a Chamfer Distance (CD) of 0.9695 m^2^, an Earth Mover’s Distance (EMD) of 1.9504 m, and a spatial angular resolution of 1.0° under the stated test conditions. Under the stated simulation assumptions, boundary analyses indicate stable reconstruction around a post-pulse-compression SNR of −8.0 dB and a conformal curvature of 12.50 m^−1^ (r=0.08 m), providing simulation-based design references for conformal radar 3D imaging.

## 1. Introduction

Millimeter-wave (mmWave) frequency-modulated continuous-wave (FMCW) radars have garnered extensive attention in aerial detection and UAV perception due to their high-resolution and all-weather capabilities [1,2]. While the two-dimensional fast Fourier transform (2D-FFT) accurately extracts range and velocity [3], achieving precise 3D point cloud imaging requires high-precision 2D direction-of-arrival (2D-DOA) estimation [4]. Traditional airborne rigid planar arrays [5,6] simplify subspace-based DOA algorithms via their ideal Vandermonde structure [7]. However, on modern high-speed aircraft, large planar arrays struggle to fit into the narrow nose radome and severely disrupt the aerodynamic profile, leading to increased drag.

Consequently, conformal arrays that seamlessly adhere to the aircraft’s surface have become essential to maximize the effective aperture within physical constraints [8,9,10]. Yet, fully curved conformal arrays suffer from severe manifold distortion, making real-time signal processing challenging [11]. To seek the optimal trade-off between hardware complexity and spatial utilization, this study constructs a “straight-curved hybrid” conformal L-shaped array. In this design, one array arm is aligned longitudinally along the aircraft fuselage, while the other arm curves circumferentially to follow the cylindrical contour of the radome. This topology preserves the low hardware redundancy of traditional L-arrays [12] while better conforming to aerodynamic boundaries.

Recent radar-system studies also show that high-resolution spatial sensing is increasingly coupled with complex propagation environments and richer electromagnetic target information. For example, RIS-assisted bistatic MIMO radar has been investigated for NLOS direction finding through covariance tensor decomposition, highlighting the need to exploit structured multi-dimensional radar data in non-line-of-sight scenarios [13]. In parallel, subterahertz radar measurement studies have emphasized full-polarization target characterization and calibration, showing that practical radar sensing accuracy depends not only on signal processing algorithms but also on front-end calibration and electromagnetic measurement fidelity [14]. These advances broaden the radar sensing context of this work: while the present study focuses on single-polarization conformal array DOA estimation and 3D point-cloud imaging, future extensions may incorporate polarization diversity, calibration compensation, and more general propagation models.

From the application perspective, real-time angle-based localization and sensing have become important in broader spatial sensing systems. Representative UWB localization studies show that antenna arrays and phase-/angle-of-arrival measurements can support centimeter-level distance and angle estimation on compact sensor nodes [15] and fully onboard relative localization without fixed infrastructure [16]. A recent review further emphasizes that real-time localization performance depends on the joint design of hardware, signal processing, and filtering pipelines [17]. These works motivate the real-time angle-sensing aspect of this paper without changing its focus: the present study addresses a conformal airborne radar scenario in which DOA estimation must additionally handle curved-array manifold distortion, coherent target echoes, and 3D point-cloud reconstruction.

Accordingly, lightweight AoA/DOA algorithms are important for real-time angle estimation under limited computational resources. Root-MUSIC converts spectral peak searching into polynomial rooting and is therefore widely used when a Vandermonde array manifold is available [18]. Reduced-dimension subspace methods reduce multidimensional spectral searches to lower-dimensional searches [19], while ESPRIT- and propagator-method-based algorithms exploit shift invariance or avoid full eigenvalue decomposition to lower the online computational burden [20]. Recent quantized and one-bit receiving architectures further reduce front-end data rates and hardware complexity [21,22]. Data-driven 2D-DOA estimation has also been investigated for array systems with constrained processing resources [23]. These methods are valuable for systems with strict hardware, power, and latency constraints. However, they are typically developed for straight, planar, or application-specific arrays and do not directly address the joint effects of conformal manifold distortion, coherent extended-target echoes, and 3D point-cloud reconstruction considered in this work.

Nevertheless, the physical arc deformation of the circumferentially curved arm disrupts the standard Vandermonde structure characteristic of a uniform linear array (ULA), causing the spatial phase differences between antenna elements to exhibit complex non-linear variations [24]. In addition, aerospace probing environments often contain multipath effects, and complex aircraft typically behave as extended targets composed of multiple dominant scattering centers distributed across the airframe. Since these scattering centers belong to the same physical entity and are often illuminated by the same radar pulse, they exhibit strong phase coherence. This leads to rank deficiency in the covariance matrix of the radar’s received data [25], which can make conventional subspace-based super-resolution DOA algorithms unreliable.

To address these challenges, existing studies have primarily focused on breakthroughs in isolated dimensions, which fail to comprehensively meet the joint processing requirements of high-speed airborne radars in complex scenarios. Regarding the mitigation of conformal array manifold distortion [26,27,28], although existing virtual array transformation techniques can map a conformal array into a virtual uniform linear array (ULA), their mathematical premises usually rely on the assumption of mutually uncorrelated signal sources. When confronted with strongly coherent echoes received by radar, the construction mechanism of traditional array transformation matrices struggles to converge. In terms of 2D-DOA estimation for coherent signals [20,29,30], extensive decoherence research has been conducted based on spatial smoothing and cross-correlation matrix reconstruction. However, the mathematical derivations of these decoupling and angle estimation algorithms for L-shaped arrays are generally founded on the strict assumption that the two array segments are “mutually orthogonal and perfectly straight.” If traditional linear L-array algorithms are directly applied to a conformal array with a curved Z-axis, the non-linear spatial phase coupling will induce severe angle estimation errors and even cause target loss. Furthermore, when dealing with dense coherent point targets, existing pairing algorithms based on 2D spatial spectral searches are highly susceptible to mismatches (Ghost Targets) at low signal-to-noise ratios (SNRs) [31]. Moreover, the two-dimensional spectral-search stage in these methods introduces a large grid-dependent computational burden, making it difficult for resource-constrained airborne computers to meet real-time requirements. In this study, the subsequent Frobenius-norm pairing is implemented as an exhaustive search only for the small number of dominant scatterers in each detected cell, and its real-time limitation is explicitly discussed with practical assignment-based alternatives in Section 2.4.5.

To address this gap, this paper proposes a joint conformal radar 3D imaging framework. The primary contributions are:

1. Virtual manifold mapping for the conformal L-shaped array. Tailored to cylindrical platforms, an asymmetric straight-curved L-shaped array model is established. An orthogonal transformation matrix is devised to compensate for non-linear phase coupling within a predefined observation sector, enabling a virtual uniform linear array (ULA) representation that supports subsequent Vandermonde-based processing.
2. Reduced-dimensionality coherent DOA estimation and robust angle pairing. Using subarray division, spatial smoothing techniques are introduced to break target coherence. By integrating the reconstructed virtual ULA with the Root-MUSIC algorithm, independent and rapid 1D polynomial root-finding is achieved, thereby circumventing the computationally exhaustive 2D spatial spectral search. Subsequently, a global cost function based on the cross-covariance Frobenius norm is formulated to reliably pair the decoupled azimuth and elevation angles, significantly suppressing spatial ghost targets.
3. 3D point cloud imaging and quantitative 3D evaluation. Based on the robustly paired 2D-DOA results, 3D point cloud imaging of coherent targets is performed. Furthermore, advanced 3D computer vision metrics (e.g., CD and HD) are introduced to systematically quantify the geometric restoration accuracy. Finally, simulation-based boundary analyses are conducted to characterize the operating limits of the algorithm concerning post-pulse-compression SNR and conformal curvature under the considered assumptions.

The remainder of this paper is organized as follows. Section 2 describes the radar-target geometry, FMCW signal model, conformal L-shaped array model, and proposed 3D imaging algorithm. Section 3 presents the simulation setup, 3D point-cloud reconstruction results, quantitative metrics, and computational complexity analysis. Section 4 discusses the robustness boundaries, practical implementation considerations, and limitations of the current simulation-based validation. Section 5 concludes the paper.

## 2. Materials and Methods

### 2.1. Radar-Target Spatial Geometry Model

**Figure 1:**
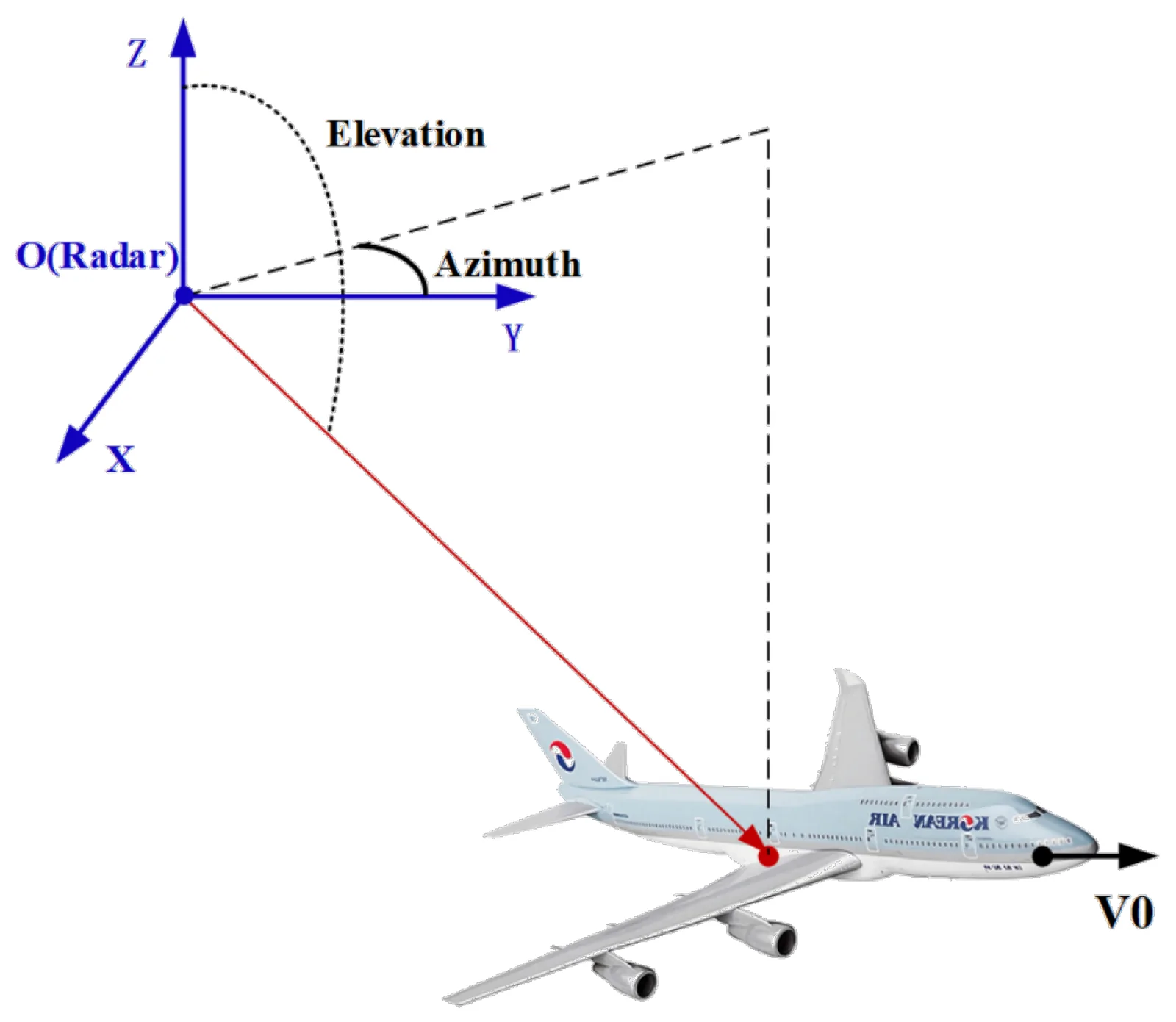
Geometric relationship of the radar detecting a moving extended target in a 3D Cartesian coordinate system.

To describe the 3D point cloud imaging scenario of the millimeter-wave (mmWave) radar, a 3D Cartesian coordinate system (x,y,z) is first established, as illustrated in Figure 1. The frequency-modulated continuous-wave (FMCW) radar, equipped with the L-shaped conformal antenna array, is deployed at the origin O(0,0,0). Suppose an extended aircraft target is present in the probing space, whose overall spatial reference is defined by its centroid *C*. The target undergoes dynamic motion with a relative velocity vector v with respect to the radar. The dotted line passing through point *C* and parallel to the z-axis intersects the XOY plane at the projection point *P*.

In Figure 1, the label $V_{0}$ denotes the target velocity vector v used in the mathematical model below; the notation is clarified here to avoid confusion between the figure label and the bold lower-case vector convention adopted in the text.

Assume there is an arbitrary scattering point P on the extended target, whose spatial position relative to the radar can be determined by the slant range *R*, azimuth angle ϕ, and elevation angle θ. In this geometric model, the elevation angle θ∈[0,π] is defined as the angle between the target direction vector and the positive Z-axis, while the azimuth angle ϕ∈[0,π] is defined as the angle between the projection of the target direction vector onto the XOY plane and the positive X-axis. Although the term “elevation” is used throughout this paper, θ is geometrically a polar/zenith-type angle measured from the positive Z-axis, consistent with the angle label in Figure 1. This definition corresponds to the front observation half-space considered in this study. Therefore, the present formulation does not attempt to resolve the ±ϕ ambiguity outside this predefined sector; extending it to full-azimuth coverage would require an atan2-type angular reconstruction and additional array information.

According to spatial geometric projection relationships, the 3D Cartesian coordinates (x,y,z) of the scattering point P can be expressed as in (1):

(1) $$\left\{\begin{array}{c} x=R\sin\theta \cos\phi ,\\ y=R\sin\theta \sin\phi ,\\ z=R\cos\theta . \end{array}\right.$$

Furthermore, considering that the extended target in space is in a state of motion, its kinematic characteristics must be introduced. Assume the 3D motion velocity vector of the target is denoted by the bold lower-case vector $v={\left[v_{x},v_{y},v_{z}\right]}^{T}$. For the radar system, the Doppler frequency shift of the echo signal is entirely determined by the target’s radial velocity $v_{r}$ along the radar’s Line-of-Sight (LoS) direction. Let the position vector of the scattering point be $p={\left[x,y,z\right]}^{T}$. By projecting the velocity vector v onto the direction of the position vector p, the radial velocity $v_{r}$ can be calculated via the inner product as shown in (2):

(2) $$v_{r}=\frac{p^{T}v}{∥p∥}=\frac{xv_{x}+yv_{y}+zv_{z}}{R}.$$

where ‖ p ‖ = *R* denotes the Euclidean norm of the position vector (i.e., the slant range from the target to the radar).

The above formulas establish the fundamental geometric mapping relationship between the physical space of the target and the radar measurement parameters. This not only elucidates the physical origins of the various parameters within the radar echo signal but also lays the theoretical foundation for subsequent array signal processing and the coordinate system reconstruction of the 3D point cloud.

### 2.2. FMCW Signal Model

This paper adopts a frequency-modulated continuous-wave (FMCW) radar with frequency center at 77 GHz, corresponding to an operating wavelength of λ≈3.896 mm. According to the standard FMCW radar principle, when the radar wave illuminates the moving target (with relative radial velocity $v_{r}$ and initial slant range $R_{0}$), the received echo undergoes digital down converter (DDC) and low-pass filtering. By neglecting minuscule higher-order terms such as the residual video phase (RVP), the intermediate frequency (IF) signal $s_{IF}\left(t,m\right)$ can be directly expressed as the classical range-Doppler two-dimensional frequency shift form [31]:

(3) $$s_{IF}\left(t,m\right)\approx A\exp\left[j2\pi \left(\frac{2\mu R_{0}}{c}t+\frac{2f_{c}v_{r}T_{c}}{c}m+\frac{2f_{c}R_{0}}{c}\right)\right].$$

where *A* is the echo amplitude; $\mu =B/T_{c}$ is the chirp slope; $T_{c}$ is the chirp duration; *t* and *m* denote the fast-time and slow-time indices, respectively.

### 2.3. Geometric Structure of Straight-Curved Hybrid L-Shaped Conformal Array

**Figure 2:**
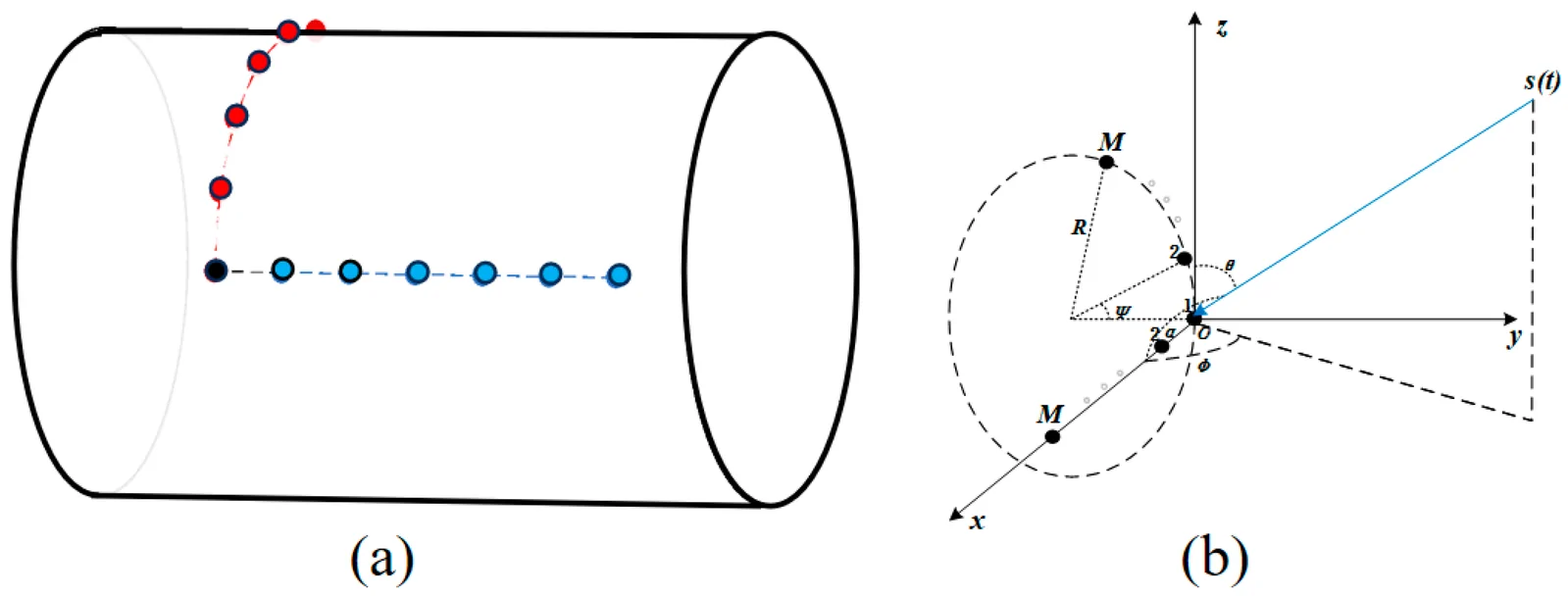
Conformal L-shaped array model. (**a**) 3D geometric model of the L-shaped conformal array on a cylindrical carrier, where the blue dots denote the straight-arm elements, the red dots denote the curved-arm elements, and the black dot denotes the shared reference element. (**b**) Spatial equivalent phase reference model, where 1 and 2 indicate representative phase-reference points used to illustrate the equivalent phase relationship.

To achieve high-precision joint estimation of 3D spatial angles (azimuth ϕ and elevation θ) on the confined surface of a high-speed cylindrical platform, this paper designs an L-shaped conformal antenna array architecture. As illustrated in Figure 2, the array comprises two orthogonally arranged subarrays: a uniform linear array (ULA) subarray deployed along the X-axis, and a conformal arc subarray deployed along the YOZ plane, conforming to the surface of the cylindrical carrier. The two subarrays are strictly orthogonal and share a common reference element located at the origin O(0,0,0) of the 3D coordinate system.

Assume the presence of a far-field narrowband coherent signal source (s(t)) in the 3D observation space, whose direction-of-arrival (DOA) is determined by the elevation angle θ∈[0,π] and the azimuth angle ϕ∈[0,π] within the predefined front observation half-space. The normalized direction cosine vector of the spatial signal can be expressed as (4):

(4) $$u\left(\theta ,\phi \right)=\left[\begin{array}{c} \sin\theta \cos\phi \\ \sin\theta \sin\phi \\ \cos\theta \end{array}\right].$$

The fundamental inter-element spacing is a half-wavelength d=λ/2. The equivalent model of the spatial phase differences generated when the radar echo signal arrives at the antenna elements at various positions is derived in detail as follows:

(1) **X-axis Uniform Linear Array (ULA) Model**

The X-axis subarray consists of $M_{x}$ receiving elements distributed in an ideal, equally spaced linear configuration [32]. The position coordinate vector of the *i*-th element ($i=0,1,\ldots ,M_{x}-1$) in space is $r_{x,i}={\left[i\cdot d,0,0\right]}^{T}$. The phase delay caused by the wave path difference of the signal arriving at this element is $\frac{2\pi }{\lambda }r_{x,i}^{T}u\left(\theta ,\phi \right)$. Consequently, the *i*-th element of the array spatial steering vector $a_{x}\left(\theta ,\phi \right)\in C^{M_{x}\times 1}$ for the X-axis ULA is given by (5):

(5) $${\left[a_{x}\left(\theta ,\phi \right)\right]}_{i}=\exp\left(j\frac{2\pi }{\lambda }id\sin\theta \cos\phi \right).$$

The steering vector of the linearly arranged subarray along the X-axis exhibits a standard Vandermonde matrix structure. The phases among its elements present a strictly linear incremental relationship, satisfying the principle of spatial shift-invariance.

(2) **YOZ Plane Conformal Arc Array Model**

As discussed in previously cited literature, to conform to the carrier’s shape and mitigate aerodynamic drag, the original linear subarray along the Z-axis is curved and conformally deployed onto the cylindrical surface, forming an arc array located in the YOZ plane. Assume this arc array contains $M_{z}$ elements, and the radius of the cylindrical carrier is *R*. To maintain the same physical arc length spacing *d* as the linear array, the central angle increment corresponding to adjacent elements is set to Δγ=d/R. Therefore, the position vector of the *k*-th arc element in the spatial coordinate system can be precisely modeled as (6):

(6) $$r_{z,k}=\left[\begin{array}{c} 0\\ R\left(\cos(k\Delta \gamma )-1\right)\\ R\sin(k\Delta \gamma ) \end{array}\right].$$

By computing the inner product between the direction vector u(θ,ϕ) and the coordinate matrix of the arc array, the steering vector $a_{z}\left(\theta ,\phi \right)\in C^{M_{z}\times 1}$ for the YOZ plane conformal arc array can be derived. Its *k*-th non-linear phase element is expressed as in (7):

(7) $${\left[a_{z}\left(\theta ,\phi \right)\right]}_{k}=\exp\left(j\frac{2\pi }{\lambda }R\left[\left(\cos(k\Delta \gamma )-1\right)\sin\theta \sin\phi +\sin\left(k\Delta \gamma \right)\cos\theta \right]\right).$$

Evidently, the steering vector $a_{z}$ of the conformal arc array contains non-linear phase terms interwoven by trigonometric functions (cos(kΔγ) and sin(kΔγ)). This geometric deformation disrupts the shift-invariance and Vandermonde structure characteristics inherent to a ULA. If one forcibly applies covariance matrix inversion or spatial smoothing directly to such manifold data, traditional subspace-based high-resolution estimation algorithms may become ineffective.

(3) **Received Signal Model for the L-Shaped Conformal Array**

In a practical multi-target detection scenario, assuming the presence of $K_{s}$ coherent signal sources, and combining the aforementioned manifold derivations with the additive environmental spatial white noise matrix N(t), the total baseband received snapshot data model for the entire L-shaped conformal array can be compactly formulated as (8):

(8) $$X\left(t\right)=\left[\begin{array}{c} X_{x}\left(t\right)\\ X_{z}\left(t\right) \end{array}\right]=\left[\begin{array}{c} A_{x}\\ A_{z} \end{array}\right]s\left(t\right)+\left[\begin{array}{c} N_{x}\left(t\right)\\ N_{z}\left(t\right) \end{array}\right].$$

where $s\left(t\right)\in C^{K_{s}\times 1}$ represents the fully coherent signal source vector transmitted (or reflected) by the $K_{s}$ targets. This joint array reception model serves as the theoretical foundation for the spatial-dimension processing discussed in the next section, namely “virtual manifold mapping” and “matrix reconstruction for decoherence”.

### 2.4. 3D Imaging Algorithm

**Figure 3:**
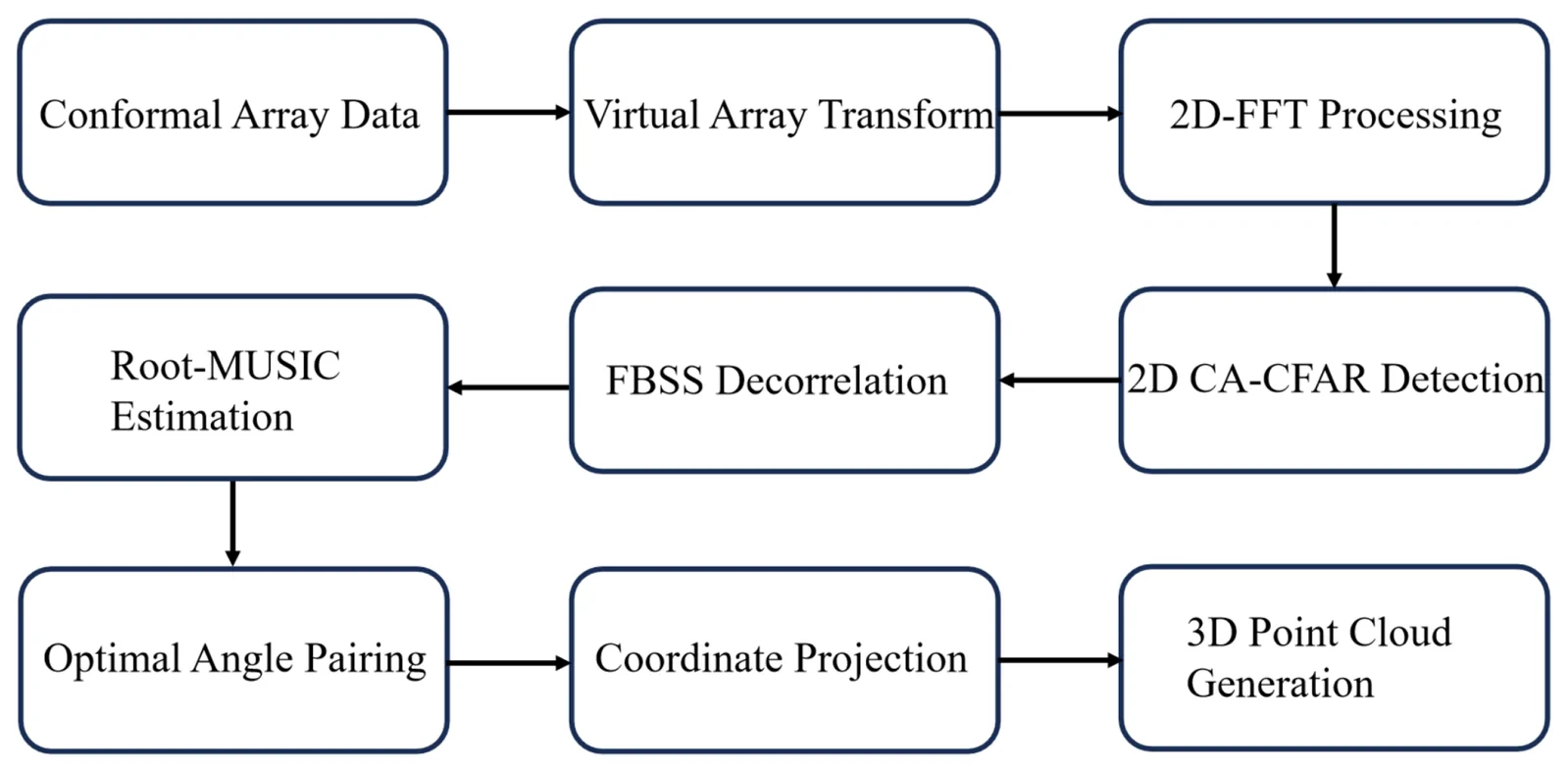
Overall block diagram of the 3D point cloud imaging algorithm based on the L-shaped conformal array.

To achieve high-precision 3D point cloud reconstruction of spatially extended targets, this paper proposes a comprehensive radar signal processing algorithm framework. The main pipeline of the proposed 3D point cloud imaging algorithm is illustrated in Figure 3.

#### 2.4.1. Virtual Array Manifold Transformation for the Conformal Array

As delineated in Section 2, the array manifold $A_{z}\left(\theta ,\phi \right)$ of the Z-axis conformal arc array contains non-linear phase terms that disrupt spatial shift-invariance. If the covariance matrix is directly calculated and the Root-MUSIC algorithm is subsequently applied, it is difficult to construct a polynomial for root-finding. Therefore, prior to performing 2D-DOA estimation, the actual conformal array manifold must be mapped into an equivalent virtual uniform linear array (ULA) via spatial interpolation or transformation techniques [24,32].

Assume that the radar’s pre-estimated observation sector for the target is defined by $\Theta =[\theta_{1},\theta_{s}]$ (elevation angle range) and $\Phi =[\phi_{1},\phi_{s}]$ (azimuth angle range). Discretized sampling is performed within this spatial sector at a specific step size to construct the actual array manifold matrix $A\in C^{M_{z}\times N_{s}}$ of the conformal array, where $N_{s}$ is the total number of spatial sampling points.

Simultaneously, a desired virtual ULA is defined (with an equivalent number of elements $M_{z}$ and identical element spacing d=λ/2). The ideal manifold matrix of this virtual ULA at the same spatial sampling points is denoted as $\bar{A}\in C^{M_{z}\times N_{s}}$.

The core philosophy of the virtual array transformation is to find a constant linear transformation matrix $G\in C^{M_{z}\times M_{z}}$ such that the mapped actual manifold approximates the ideal virtual ULA manifold within the given observation sector, satisfying (9):

(9) $$G^{H}A\approx \bar{A}.$$

The normalized mapping error used later in the curvature analysis is defined as

$$\varepsilon_{\mathrm{map}}=\frac{{∥TA-\bar{A}∥}_{F}}{{∥\bar{A}∥}_{F}}.$$

This dimensionless quantity measures the residual manifold mismatch after the unitary virtual transformation over the predefined observation sector.

To minimize the mapping error, an objective function $\min_{G}{\left\|G^{H}A-\bar{A}\right\|}_{F}^{2}$ can be constructed based on the Least Squares (LS) criterion, where ${\left\|\;\cdot \;\right\|}_{F}$ denotes the Frobenius norm. Solving this optimization problem yields the preliminary transformation matrix G as shown in (10):

(10) $$G={\left(AA^{H}\right)}^{-1}A{\bar{A}}^{H}.$$

However, directly using matrix G for left-multiplying and mapping the received data will alter the statistical characteristics of the spatial white Gaussian noise in the original array receiver, turning it into colored noise, thereby severely degrading the subsequent subspace-based DOA estimation performance. To preserve the spatial whitening characteristics of the noise (i.e., satisfying the unitary matrix property of the transformation matrix, $T^{H}T=I$), G must be orthogonalized. This paper employs optimal approximation under orthogonal constraints to construct the final transformation matrix T in (11):

(11) $$T={\left(G^{H}G\right)}^{-1/2}G^{H}.$$

After acquiring this mapping matrix, a linear transformation is applied to the multi-channel snapshot data matrix $X_{z}\left(t\right)$ actually received by the YOZ plane conformal array, as defined in (12):

(12) $${\tilde{X}}_{z}\left(t\right)=TX_{z}\left(t\right).$$

**Figure 4:**
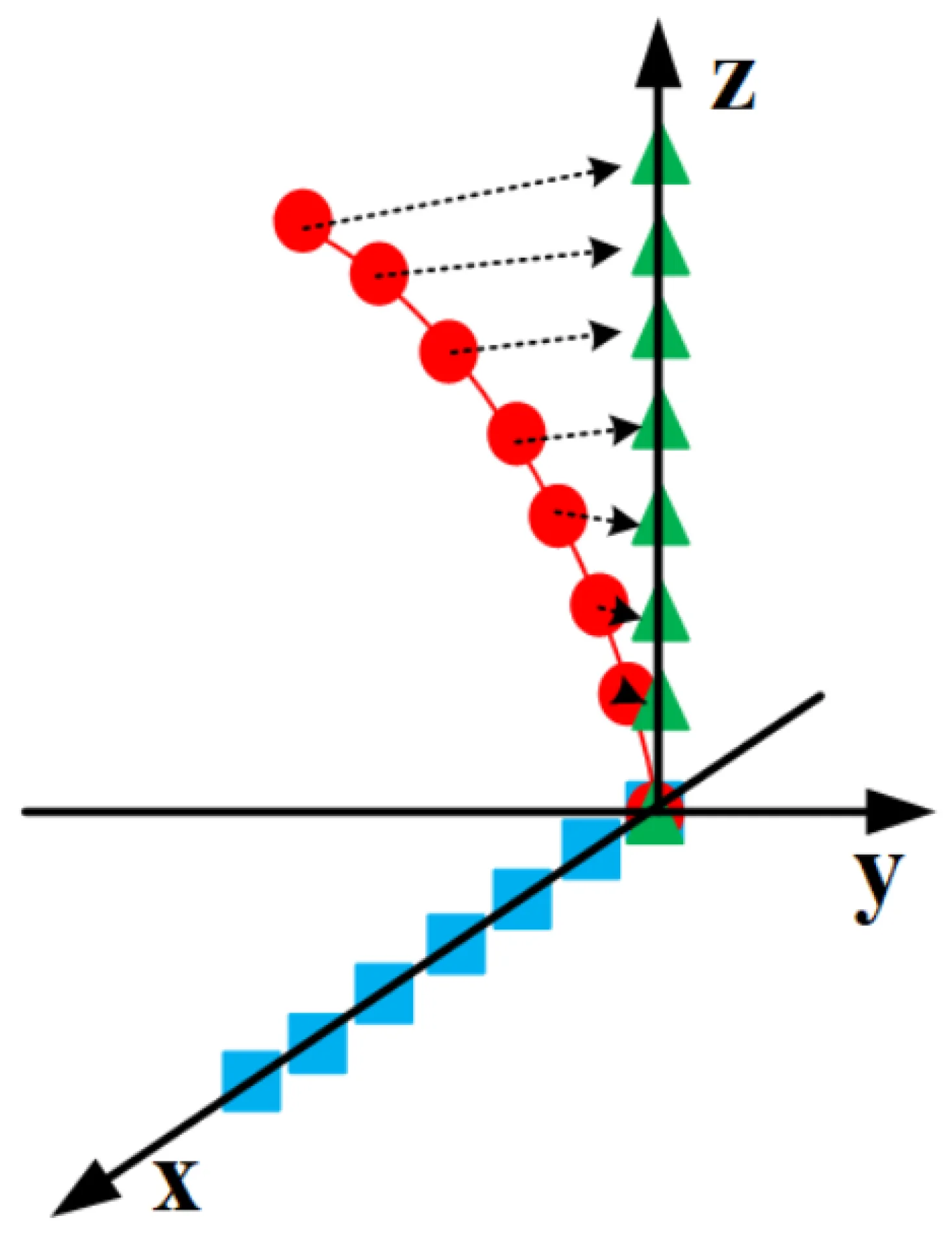
Virtual manifold transformation of the YOZ plane conformal arc array, where the colored symbols represent sampled manifold points before and after virtual mapping.

Following the mapping in the above equation, the newly generated data matrix ${\tilde{X}}_{z}\left(t\right)$ approximates the received data of an ideal ULA with a standard Vandermonde structure within the predefined observation sector. Therefore, the non-linear phase distortion introduced by the arc array can be compensated to a large extent rather than removed globally. The accuracy of this mapping depends on the sector width, angular sampling density, curvature, array calibration, element position accuracy, element pattern consistency, and mutual coupling level. A comparison of the array spatial topologies and the equivalent mapping effect before and after the transformation is presented in Figure 4.

#### 2.4.2. 2D Range-Doppler FFT and CA-CFAR Detection

After acquiring the raw IF data, a standard 2D-FFT is applied to the fast-time and slow-time dimensions to extract the range and Doppler information. To improve the SNR and prevent energy misdetection, multi-channel non-coherent integration is performed to generate a global Range-Doppler Map (RDM) as defined in (13):

(13) $$\mathrm{RDM}\left(k,l\right)=\sum_{i=1}^{M_{\mathrm{total}}}{\left|Y_{i}\left(k,l\right)\right|}^{2}.$$

Subsequently, the well-established 2D Cell-Averaging Constant False Alarm Rate (2D CA-CFAR) [33] is employed to adaptively extract valid scattering centers from the strong background clutter. When the power of a cell exceeds the adaptive threshold, it is determined that a valid target exists, yielding the target detection cells $\left(k_{d},l_{d}\right)$. The complex frequency-domain values are then extracted to construct a Single Snapshot Observation Vector.

#### 2.4.3. Coherent DOA Estimation Based on FBSS and Root-MUSIC

**Figure 5:**
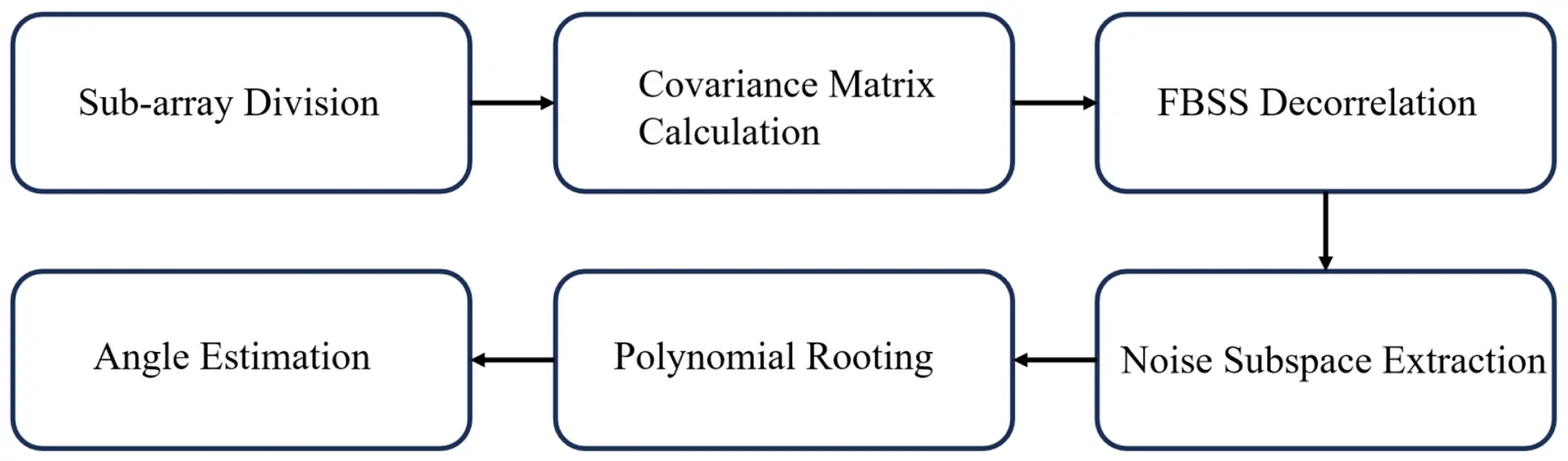
Flowchart of the coherent DOA estimation based on FBSS and Root-MUSIC.

To address the severe rank deficiency problem of the array covariance matrix caused by highly coherent scattering centers falling within the same resolution cell, a cascaded decoherence and angle estimation algorithm is proposed. The overall implementation pipeline of this processing framework is illustrated in Figure 5.

As depicted in the flowchart, following the virtual manifold mapping in (12), both the physical X-axis and the equivalent Z-axis subarrays possess a standard Vandermonde structure. According to the Forward-Backward Spatial Smoothing (FBSS) theory [34,35], the array is first divided into multiple overlapping sub-arrays. After computing the forward smoothed covariance matrix $R_{f}$ by averaging the sub-array matrices, an exchange matrix J is introduced to execute conjugate reversal. The full-rank FBSS augmented matrix $R_{fb}$ is thus reconstructed as in (14):

(14) $$R_{fb}=\frac{1}{2}\left(R_{f}+JR_{f}^{*}J\right).$$

After acquiring the decorrelated and full-rank covariance matrix $R_{fb}$, an eigenvalue decomposition (EVD) is performed to extract the signal subspace and the noise subspace $U_{n}$, as formulated in (15):

(15) $$R_{fb}=U_{s}\Lambda_{s}U_{s}^{H}+U_{n}\Lambda_{n}U_{n}^{H}.$$

The EVD in (15) also indicates that source-number selection is a necessary part of the subsequent subspace construction. In the controlled simulations of this study, the number of dominant coherent scattering centers within each detected cell is prescribed as $K_{s}=2$ and the FBSS subarray length is set to L=5 for each eight-element subarray. This satisfies the resolvable-subspace requirement $K_{s}<L$ and keeps the model order within the effective aperture available after smoothing. The detected 3D point count reported later is accumulated over many RDM cells and should not be interpreted as the number of coherent sources within one cell. These fixed values allow the conformal-array mapping, FBSS decorrelation, and Root-MUSIC imaging pipeline to be evaluated under repeatable conditions. In practical airborne detection, however, this number is generally unknown and may vary with target aspect, SNR, scattering strength, CFAR cell grouping, and multipath/clutter. Therefore, before determining the signal subspace dimension and selecting the $K_{s}$ roots closest to the unit circle in Root-MUSIC, a source-enumeration step should be included. Classical information-theoretic criteria such as AIC and MDL can estimate the model order from the eigenvalue distribution of the reconstructed covariance matrix, while eigenvalue-gap or noise-floor threshold rules provide simpler engineering alternatives [36]. Underestimating the source number may merge weak scattering centers into the noise subspace, whereas overestimating it may introduce noise roots and enlarge the angle-pairing problem. Both cases can degrade Root-MUSIC rooting and the subsequent Frobenius-norm-based pairing.

To avert the high-complexity spatial spectral peak search over the predefined observation sector, the Root-MUSIC algorithm [18] is used, transforming the spatial spectral search into a polynomial root-finding problem. Let *q* denote the dimension of the steering polynomial vector and let $b\left(z\right)={\left[1,z,\ldots ,z^{q-1}\right]}^{T}$. The root-finding polynomial function f(z) is constructed in (16):

(16) $$f\left(z\right)=z^{q-1}b^{T}\left(z^{-1}\right)U_{n}U_{n}^{H}b\left(z\right)=0.$$

Solving (16) yields the characteristic complex roots. By sorting their magnitudes and filtering out the $K_{s}$ valid signal roots closest to the unit circle, the corresponding independent angle-of-arrival variables for the Z-axis and X-axis are directly calculated via the inverse trigonometric functions in (17) and (18):

(17) $$\theta_{z,k}=\arccos\left(\frac{\lambda }{2\pi d}\arg\left(Z_{z,k}\right)\right).$$

(18) $$\alpha_{x,k}=\arccos\left(\frac{\lambda }{2\pi d}\arg\left(Z_{x,k}\right)\right).$$

It is essential to point out that, because an orthogonal, independent L-shaped conformal array is employed, $\theta_{z,k}$ obtained from (17) is precisely the target’s true elevation angle (Elevation $\theta_{k}$). However, $\alpha_{x,k}$ derived from (18) for the X-axis is merely a cone angle component (Cone Angle $\alpha_{x}$), as specifically depicted in Figure 2b. Furthermore, the multiple target angles estimated independently by the two axes are in a state of order ambiguity. How to achieve the precise pairing of these independent parameters and subsequently compute the true azimuth angle ($\phi_{k}$) will be the focal point of the next section.

#### 2.4.4. Angle Pairing and 3D Point Cloud Generation

In multi-target complex scenarios, executing the Root-MUSIC algorithm independently on the X-axis subarray and the Z-axis virtual subarray yields two independent and unordered sets of angles: the cone angle component set $\alpha_{x}=\left\{\alpha_{x,1},\alpha_{x,2},\ldots ,\alpha_{x,K_{s}}\right\}$ and the elevation angle set $\theta_{z}=\left\{\theta_{z,1},\theta_{z,2},\ldots ,\theta_{z,K_{s}}\right\}$. If combined directly, this will induce severe order ambiguity, leading to the emergence of false spatial targets (i.e., the ghost target problem).

Because the X-axis and Z-axis subarrays share a reference element located at the origin, the spatial cross-correlation information of the respective scattering centers is inherently embedded within the received data of both axes. To achieve the automatic one-to-one correspondence of these $K_{s}$ multi-target angles, this paper proposes an optimal angle pairing algorithm based on the minimization of the Frobenius norm of the cross-covariance matrix.

First, using the two sets of unordered angles estimated by Root-MUSIC in the previous step, the corresponding estimated X-axis array manifold matrix ${\hat{A}}_{x}$ and estimated Z-axis array manifold matrix ${\hat{A}}_{z}$ are reconstructed. Subsequently, each of the two subarrays is further divided into front and rear halves (i.e., ${\hat{A}}_{x1},{\hat{A}}_{x2}$ and ${\hat{A}}_{z1},{\hat{A}}_{z2}$). Using the received data $X_{x}$ and $X_{z}$, the auto-covariance matrices $R_{xx},R_{zz}$ and cross-covariance matrices $R_{xz},R_{zx}$ are computed. The corresponding front-rear sub-block covariance matrices $R_{z1z2}$ and $R_{x1x2}$ are obtained from the partitioned Z-axis and X-axis subarray snapshots.

Define an unknown global permutation matrix C with dimensions of $K_{s}\times K_{s}$. According to the equivalent principle of spatial signal cross-correlation, the correct arrangement order should render the signal source covariance matrices—derived based on both auto-correlation and cross-correlation—highly consistent. To this end, we construct the following cost function with respect to the independent variable *C*, as expressed in (19):

(19) $$J\left(C\right)={∥R_{s1}-CR_{s2}∥}_{F}+{∥CR_{s3}-R_{s4}∥}_{F}.$$

where ${\left\|\;\cdot \;\right\|}_{F}$ denotes the Frobenius norm of a matrix. $R_{s1}$ to $R_{s4}$ are the projections of the respective covariance matrices calculated using the Moore–Penrose pseudoinverse (denoted by the superscript +), as defined in (20):

(20) $$\begin{array}{c} R_{s1}={\hat{A}}_{z1}^{+}R_{z1z2}{\left({\hat{A}}_{z2}^{+}\right)}^{H},\\ R_{s2}={\hat{A}}_{x}^{+}R_{xz}{\left({\hat{A}}_{z}^{+}\right)}^{H},\\ R_{s3}={\hat{A}}_{x1}^{+}R_{x1x2}{\left({\hat{A}}_{x2}^{+}\right)}^{H},\\ R_{s4}={\hat{A}}_{z}^{+}R_{zx}{\left({\hat{A}}_{x}^{+}\right)}^{H}. \end{array}$$

In the present simulations, the number of dominant scattering centers in each detected cell is small; therefore, all possible $K_{s}!$ permutation matrices are traversed to obtain the global minimum of the cost function in (19). This exhaustive search is useful for validating the proposed Frobenius-norm pairing criterion because it avoids local assignment ambiguities. However, its factorial complexity is not suitable for dense multi-target scenes in which $K_{s}$ may increase to dozens. For such real-time airborne applications, the exhaustive search should be replaced by an assignment-based implementation: coarse angular or range-Doppler gating can first restrict the candidate pairs, and the remaining row-wise covariance-mismatch costs can then be solved using a polynomial assignment algorithm, such as the Hungarian algorithm [37]. The exhaustive form used in this study yields $C_{\mathrm{opt}}$ as shown in (21):

(21) $$C_{\mathrm{opt}}=\arg\min_{C}J\left(C\right).$$

The optimal permutation matrix $C_{\mathrm{opt}}$ precisely records the true correspondence between the cone angles and elevation angles. By applying this permutation matrix, the initially disordered $\theta_{z}$ can be seamlessly paired with $\alpha_{x}$.

Upon the completion of pairing, assume the correctly paired combination for the *k*-th target point is $\left(\alpha_{x,k},\theta_{z,k}\right)$. At this juncture, $\theta_{z,k}$ is precisely the true elevation angle $\theta_{k}$. According to the projection theorem in 3D solid geometry, the true azimuth angle $\phi_{k}$ of the target can be calculated through the non-linear conversion between the cone angle and the elevation angle, as shown in (22). Because the inverse-cosine operation returns the principal value, this conversion is valid for the front half-space observation sector ϕ∈[0,π] defined in the geometric model.

(22) $$\phi_{k}=\arccos\left(\frac{\cos(\alpha_{x,k})}{\sin(\theta_{k})}\right).$$

Here, $R_{z1z2}$ and $R_{x1x2}$ denote the off-diagonal sub-block covariance matrices between the front and rear halves of the equivalent Z-axis and X-axis subarrays, respectively. Using these sub-blocks makes the matrix products in (20) dimensionally conformable and keeps the two auto-correlation projections symmetric.

Thus far, the algorithm has obtained the distance $R_{d}$, the true azimuth angle $\phi_{k}$, and the true elevation angle $\theta_{k}$ for all coherent scattering centers within the target detection cell *d*. Utilizing the mapping formula from spherical polar coordinates to 3D Cartesian coordinates established in Section 2.1, the Cartesian coordinates $\left(x_{k},y_{k},z_{k}\right)$ are obtained via (23):

(23) $$\left[\begin{array}{c} x_{k}\\ y_{k}\\ z_{k} \end{array}\right]=\left[\begin{array}{c} R_{d}\sin\theta_{k}\cos\phi_{k}\\ R_{d}\sin\theta_{k}\sin\phi_{k}\\ R_{d}\cos\theta_{k} \end{array}\right].$$

By projecting the coordinate set of all targets detected in the radar’s field of view into 3D space, 3D point-cloud imaging of complex extended targets is ultimately accomplished.

#### 2.4.5. Computational Complexity and Real-Time Considerations

Because real-time processing is a key motivation of the proposed framework, the main computational cost of each processing block is summarized in Table 1. Let $N_{r}$ and $N_{d}$ denote the numbers of range and Doppler samples, respectively; *M* denotes the number of receiving channels; $K_{s}$ denotes the number of coherent sources within a detected cell; *L* denotes the subarray length used for FBSS; and $N_{s}$ denotes the number of angular grid samples used offline to construct the virtual manifold transformation. The proposed method avoids a two-dimensional spectral peak search over $N_{\theta }N_{\phi }$ angle grids by converting the DOA estimation into two one-dimensional Root-MUSIC polynomial rooting problems after virtual manifold transformation and FBSS.

To complement the complexity analysis with measured evidence, we also profiled the main online processing blocks in MATLAB R2025a on an AMD Ryzen 7 5800H CPU with 16 GB RAM. The profiling used the simulation configuration of 300 range samples, 128 Doppler chirps, two eight-element algorithmic subarray snapshots, and 20 representative detected cells, and each block was repeated 30 times. These parameters are the same as the radar and algorithm settings summarized later in Section 3; they are stated here as a forward reference to the simulation setup because the runtime evidence is discussed before the Section 3. The offline construction of the virtual manifold transformation matrix was excluded, while the online matrix-vector transformation was included. The CA-CFAR detector was implemented in an equivalent vectorized form for engineering-style MATLAB profiling.

The accumulated mean runtime of the profiled online blocks is about 45.2 ms for the tested MATLAB configuration with 20 representative detected cells. Since the representative imaging example contains 138 recovered scattering centers accumulated over all detected RDM cells, this value should be interpreted as a per-20-cell profiling result rather than the runtime of the full frame. A simple linear extrapolation of the cell-wise blocks gives approximately 221 ms for 138 cells, while the FFT and CA-CFAR blocks remain frame-level costs. The relatively large 11.31 ms online virtual-manifold transformation time is dominated by MATLAB loop/function-call overhead in the non-vectorized profiling script; the underlying arithmetic is only a small matrix-vector multiplication and can be further reduced by vectorized or compiled implementation. The results indicate that the DOA-related blocks, including FBSS, Root-MUSIC, and angle pairing, remain moderate compared with the FFT and online virtual-array transformation stages.

To further compare the DOA estimation core, we profiled three representative two-dimensional DOA estimators in MATLAB R2025a on an AMD Ryzen 7 5800H CPU with 16 GB RAM. For a fair algorithm-level comparison, all methods were evaluated using three sources, 200 snapshots, 20 dB SNR, and an eight-element array scale. The proposed method was timed as the two-axis 2D Root-MUSIC estimation and Frobenius-norm-based angle-pairing core without FBSS preprocessing, because the benchmark implementations of 2D-MUSIC and Unitary-ESPRIT also follow their standard non-FBSS processing pipelines. This separate benchmark is an algorithm-level comparison under the snapshot regime used by the available baseline implementations; it is not intended to represent the single-snapshot per-detected-cell deployment used in the 3D imaging pipeline. Each method was repeated 30 times, and the average and median runtimes are reported in Table 2.

The measured results show that the proposed 2D Root-MUSIC core is approximately 27.6 times faster than the conventional L-shaped 2D-MUSIC baseline in terms of average runtime. Its runtime is also comparable to that of 2D Unitary-ESPRIT, while retaining compatibility with the proposed virtual-array and Frobenius-norm angle-pairing framework. These results support the claim that avoiding exhaustive 2D spatial spectral search substantially reduces the online DOA processing burden.

The transformation matrix T is computed offline for a predefined sector and therefore does not dominate the per-frame online complexity. The exhaustive permutation search used for angle pairing is suitable for the small number of dominant coherent scatterers considered in this study and provides a globally optimal pairing benchmark for the proposed cost function. Nevertheless, its $O(K_{s}!K_{s}^{3})$ complexity becomes a bottleneck for dense multi-target scenes. In practical real-time implementations, this block should be accelerated by restricting candidate pairs using coarse angular or range-Doppler gating, or by replacing exhaustive enumeration with assignment-based optimization such as the Hungarian algorithm, whose standard implementation has polynomial complexity. This limitation is further discussed in Section 4.2.

#### 2.4.6. Evaluation Metrics for 3D Point Cloud Reconstruction

After completing the non-linear calculation of 3D spatial coordinates, to quantify the reconstruction accuracy of the proposed radar 3D imaging algorithm, this paper introduces four core quantitative evaluation metrics universally recognized in the fields of 3D computer vision and high-resolution radar imaging [38,39,40,41]: Chamfer Distance (CD), Hausdorff Distance (HD), Earth Mover’s Distance (EMD), and the comprehensive metric (F-Score).

Let the predicted point cloud set estimated by the radar system be denoted as $S_{1}$ (with a total of $|S_{1}|$ points), and the ground truth point cloud set representing the target’s actual spatial geometric physical model be denoted as $S_{2}$ (with a total of $|S_{2}|$ points).

(1) **Chamfer Distance (CD)**

Chamfer Distance is used to measure the average closest squared distance between two point sets, which can effectively gauge the global overall similarity between the predicted point cloud and the ground truth. It is composed of an accuracy term ($S_{1}\to S_{2}$) and a completeness term ($S_{2}\to S_{1}$), defined in (24):

(24) $$d_{CD}\left(S_{1},S_{2}\right)=\frac{1}{|S_{1}|}\sum_{x\in S_{1}}\min_{y\in S_{2}}{∥x-y∥}_{2}^{2}+\frac{1}{|S_{2}|}\sum_{y\in S_{2}}\min_{x\in S_{1}}{∥y-x∥}_{2}^{2}.$$

(2) **Hausdorff Distance (HD)**

Hausdorff Distance measures the maximum degree of mismatch between two point sets and is primarily used to evaluate the algorithm’s outlier error under worst-case scenarios. It can effectively verify the angle pairing algorithm’s capability to suppress ghost targets. The bidirectional HD is defined in (25):

(25) $$H\left(S_{1},S_{2}\right)=\max\left(h\left(S_{1},S_{2}\right),h\left(S_{2},S_{1}\right)\right).$$

where the unidirectional distance is expressed as $h\left(S_{1},S_{2}\right)={max}_{x\in S_{1}}{min}_{y\in S_{2}}{\left\|x-y\right\|}_{2}$.

(3) **Earth Mover’s Distance (EMD)**

Earth Mover’s Distance is employed to compare the global spatial distribution similarity of two 3D point clouds. Its core philosophy is to find a bijection mapping $\varphi :S_{1}\to S_{2}$ to minimize the average distance cost between corresponding points in the two sets. The mathematical definition of EMD is given by (26):

(26) $$\mathrm{EMD}\left(S_{1},S_{2}\right)=\min_{\varphi :S_{1}\to S_{2}}\frac{1}{|S_{1}|}\sum_{x\in S_{1}}{∥x-\varphi \left(x\right)∥}_{2}.$$

Unlike CD, which can process point sets of different sizes, the standard EMD algorithm requires that the two point cloud sets undergoing evaluation possess an equal number of points, i.e., $|S_{1}|=|S_{2}|$. In practical radar system evaluations, because the number of coherent scattering centers extracted by radar is typically fewer than the number of points in a high-density ground truth physical model, the algorithm equalizes the point counts through multiple random downsampling operations. Subsequently, it computes the optimal match and takes the average of multiple calculations as the final EMD evaluation metric.

(4) **F-Score**

Chamfer Distance is highly susceptible to the influence of point cloud density, whereas the F-score can evaluate point cloud quality in a more balanced manner. The F-score is the harmonic mean of Precision and Recall, enabling a comprehensive evaluation of the accuracy and completeness of the 3D point cloud reconstruction results. Given a distance tolerance threshold *d*, the Precision P(d) and Recall R(d) for the set $S_{1}$ are defined in (27) and (28), respectively:

(27) $$P\left(d\right)=\frac{1}{|S_{1}|}\left|\left\{x\in S_{1}:\min_{y\in S_{2}}∥x-y∥<d\right\}\right|.$$

(28) $$R\left(d\right)=\frac{1}{|S_{2}|}\left|\left\{y\in S_{2}:\min_{x\in S_{1}}∥x-y∥<d\right\}\right|.$$

where ∣ · ∣ denotes the cardinality of a set (i.e., the number of elements within the set). Based on the aforementioned definitions, the comprehensive evaluation metric F-score for the point cloud is defined in (29):

(29) $$F\text{-}\mathrm{score}\left(d\right)=\frac{2\cdot P(d)\cdot R(d)}{P(d)+R(d)}.$$

## 3. Results

### 3.1. 3D Point Cloud Imaging Verification of Complex Coherent Targets

To validate the proposed L-shaped conformal array 3D imaging algorithm, simulations were conducted in MATLAB. This section presents the range-Doppler processing, virtual manifold mapping, coherent DOA estimation, and final 3D point cloud reconstruction.

(1) **Simulation Parameter Settings and Target Model**

This paper simulates a 77 GHz FMCW mmWave radar system. The primary simulation parameters of the radar system are listed in Table 3.

**Figure 6:**
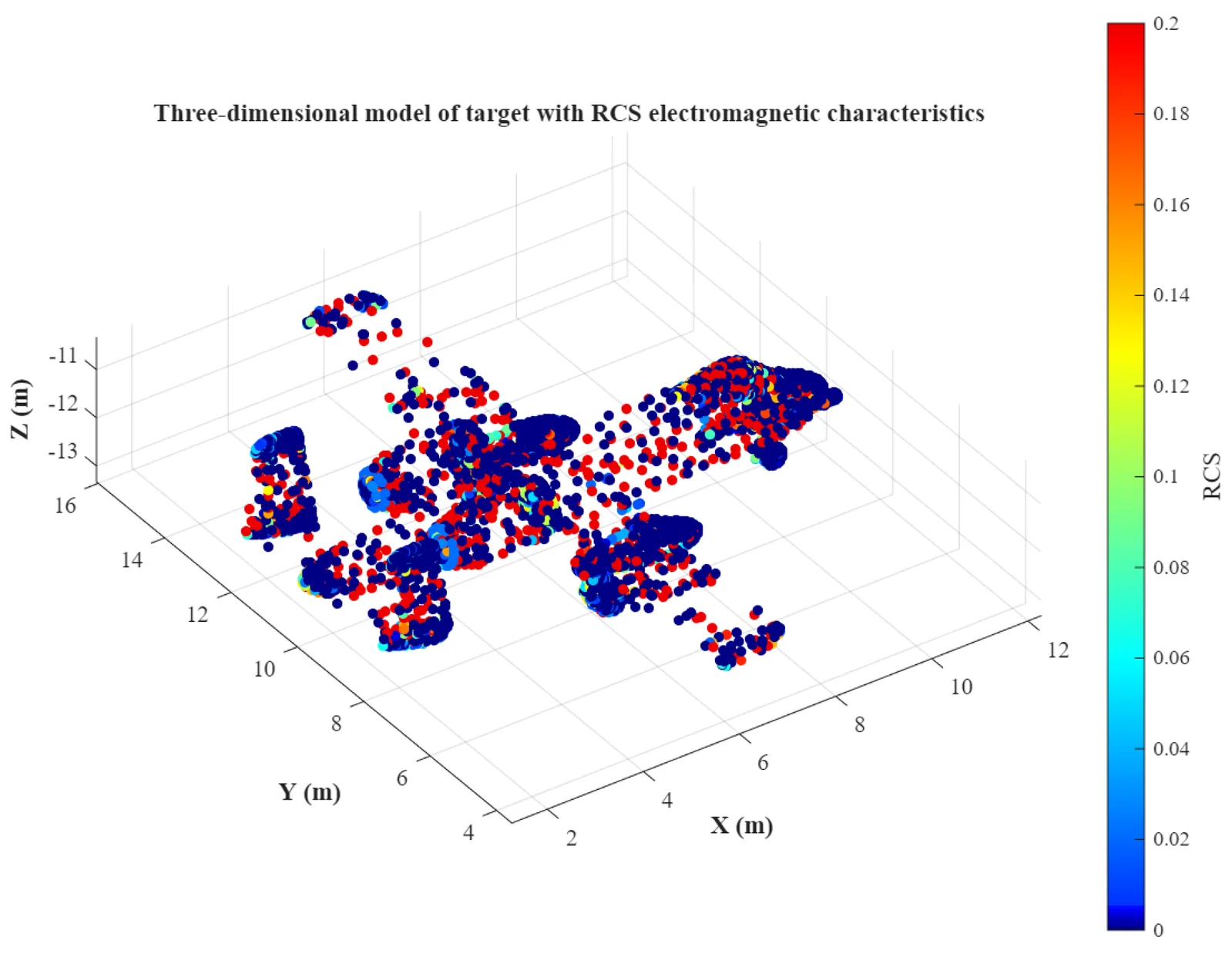
Aircraft model composed of scattering centers.

Regarding the target model, this paper imports a 3D discrete aircraft model composed of strongly coherent scattering centers. As illustrated in Figure 6, the model defines the precise geometric topology of the aircraft, with corresponding radar cross-section (RCS) amplitudes assigned to each point (the color transition from cool to warm represents an increase in RCS intensity). The initial radial distance is set to $R_{0}=15$ m, moving with a velocity vector $v={\left[20,0,0\right]}^{T}$ m/s. With B=1.8 GHz, $T_{c}=60\;\mu$s, and $f_{s}=5$ MHz, the chirp slope is $\mu =B/T_{c}=3.0\times 10^{13}$ Hz/s and the maximum unambiguous range is $R_{\max}=cf_{s}/\left(2\mu \right)=25$ m; therefore, the selected 15 m operating range is consistent with the RDM and 3D point-cloud figures. This near-range geometry is intended to represent a proximity detection scenario for validating the conformal-array DOA and 3D point-cloud reconstruction pipeline under the adopted FMCW parameter set, rather than a conventional long-range airborne imaging scenario. Additive white Gaussian noise (AWGN) is injected at the receiving end. The representative nominal setting is a post-pulse-compression SNR of 10 dB.

The simulation target parameters are detailed in Table 4.

(2) **Conformal Array Construction and Virtual Array Manifold Transformation**

The receiver employs the straight-curved hybrid L-shaped conformal antenna array architecture described previously. Specifically, the array comprises two orthogonal sub-branches, each consisting of 8 antenna elements with an inter-element spacing of λ/2. The X-axis subarray is deployed as a standard uniform linear array (ULA). In contrast, the YOZ plane subarray is conformally distributed along a cylindrical arc with a physical radius of r=0.1 m. This operating radius corresponds to a curvature of $\kappa =10\;m^{-1}$, which remains on the stable side of the simulation-based boundary $\kappa \approx 12.50\;m^{-1}$ identified in Section 4.1, but with only a moderate margin.

According to the proposed virtual array transformation theory, the orthogonal mapping matrix T is applied directly to the original spatial snapshot matrix of the Z-axis subarray, as shown in Figure 4. Simulation results indicate that the transformed manifold accurately approximates the ideal Vandermonde structure. This step mitigates the non-linear phase distortion caused by the arc array within the predefined observation sector while approximately preserving the spatial whitening characteristics of the background noise, establishing a practical data foundation for subsequent 2D DOA estimation.

(3) **2D Range-Doppler Processing and CFAR Detection**

Following the virtual array transformation, the system executes a 2D-FFT to extract range and radial velocity information. Non-coherent integration is performed across all channels to maximize the SNR. Thereafter, the 2D CA-CFAR detector (with false alarm probability $P_{fa}=10^{-3}$) is employed to adaptively extract valid scattering centers from the background clutter.

**Figure 7:**
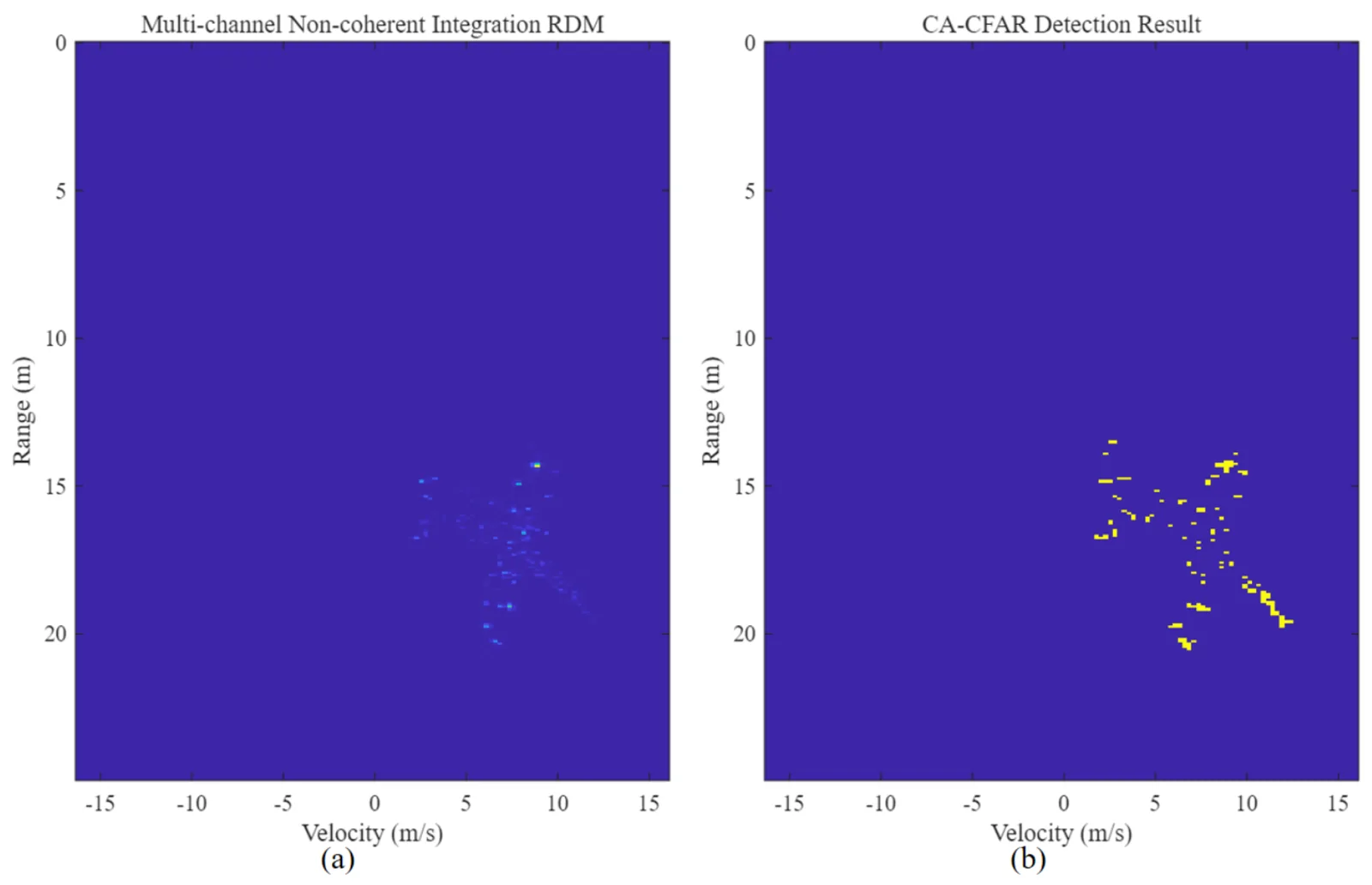
Multi-channel non-coherent integration and target detection. (**a**) Range-Doppler Map (RDM), where warmer colors indicate stronger echo intensity. (**b**) 2D CA-CFAR binarized detection results, where highlighted pixels denote detected target cells.

Figure 7a displays the Range-Doppler Map (RDM). Due to the substantial physical dimensions of the aircraft, strong scattering centers across the wings, fuselage, and tail exhibit minute range and radial velocity differences. Consequently, the target’s energy disperses across multiple adjacent range and Doppler cells, outlining its macroscopic contour. Figure 7b delineates the 2D CA-CFAR binary detection map. The algorithm effectively filters out the background noise floor while preserving the discrete spatial distribution of the aircraft. For the adopted chirp duration, the maximum unambiguous radial velocity is $v_{\max}=\lambda /\left(4T_{c}\right)\approx 16.2$ m/s. Although the target speed magnitude is 20 m/s, the radial component in the simulated aspect is approximately 7 m/s and therefore remains within this bound, so Doppler aliasing is not induced in this case. This statement is aspect-dependent: if the platform-target geometry produces a radial velocity component exceeding $v_{\max}$, Doppler ambiguity would occur and a larger chirp repetition interval margin or Doppler ambiguity-resolution strategy would be required. For each detected cell in the valid 2D index set $D={\left\{\left(k_{d},l_{d}\right)\right\}}_{d=1}^{N_{D}}$, the corresponding radial range $R_{d}$, radial velocity $v_{rd}$, and multi-channel spatial snapshot vector are extracted as inputs for the subsequent Root-MUSIC DOA estimation. Because the two eight-element arms share one physical reference antenna, the hardware contains 15 RF receiving channels. In the algorithmic L-shaped array model, the shared reference sample is copied into both subarray vectors, so $X_{x}$ and $X_{z}$ are each treated as eight-element snapshots for shift-invariant processing; this bookkeeping does not imply a duplicated RF channel.

(4) **Coherent DOA Estimation and 3D Point Cloud Reconstruction**

To address the rank deficiency caused by strongly coherent scattering centers, the algorithm independently executes FBSS on both the X-axis and the equivalent Z-axis ULA. Subsequently, the Root-MUSIC algorithm is used. By solving the polynomial and extracting the characteristic roots closest to the unit circle, the algorithm filters out noise roots, bypassing the computationally exhaustive spatial spectral search.

To evaluate the spatial resolution limit of the proposed algorithm when processing dense coherent scattering centers, a dual-target angular resolution test was conducted. In the simulation, two strongly coherent signal sources were configured, and their angular separation (Δθ) was gradually reduced from 15° down to 0°. The Root Mean Square Error (RMSE) of the angle estimation was statistically calculated over 500 Monte Carlo trials at the same 10 dB post-pulse-compression SNR as the main imaging simulation.

**Figure 8:**
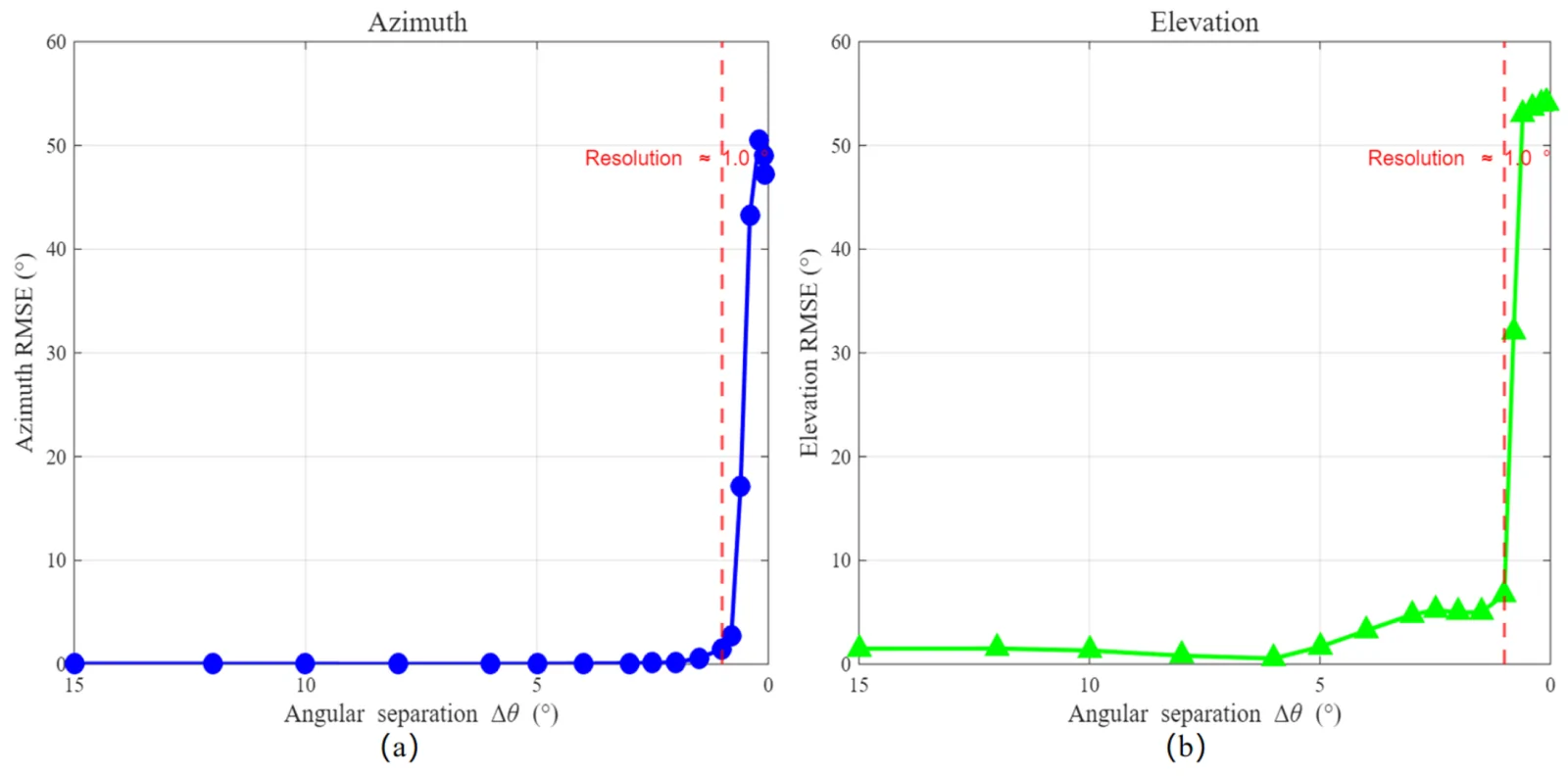
Angular resolution critical test curve for DOA estimation. (**a**) Azimuth resolution test. (**b**) Elevation angle resolution test.

As illustrated in Figure 8, the error curves for both the X-axis and equivalent Z-axis exhibit a clear transition regarding the angular separation Δθ. For Δθ>1.0°, the RMSE remains low but nonzero, indicating stable estimation under the adopted assumptions. However, when Δθ<1.0°, noise subspace aliasing causes the RMSE to increase. This indicates an angular resolution of Δθ≈1.0° in this controlled two-source simulation at a post-pulse-compression SNR of 10 dB. This value should be interpreted as an empirical Root-MUSIC super-resolution result under the adopted coherent-source model and array calibration assumptions, not as the Rayleigh beamwidth of the eight-element physical aperture. The plotted RMSE floor remains small but nonzero, and a dedicated CRB comparison will be included in future statistical performance studies. Using the extracted roots, unordered cone angles $\alpha_{x}$ and elevation angles $\theta_{z}$ are computed.

By minimizing the global cross-covariance cost function J(C), the optimal permutation matrix $C_{\mathrm{opt}}$ is obtained to pair the X-axis and Z-axis angles. The true azimuth angle $\phi_{k}$ is then derived via inverse cosine projection.

**Figure 9:**
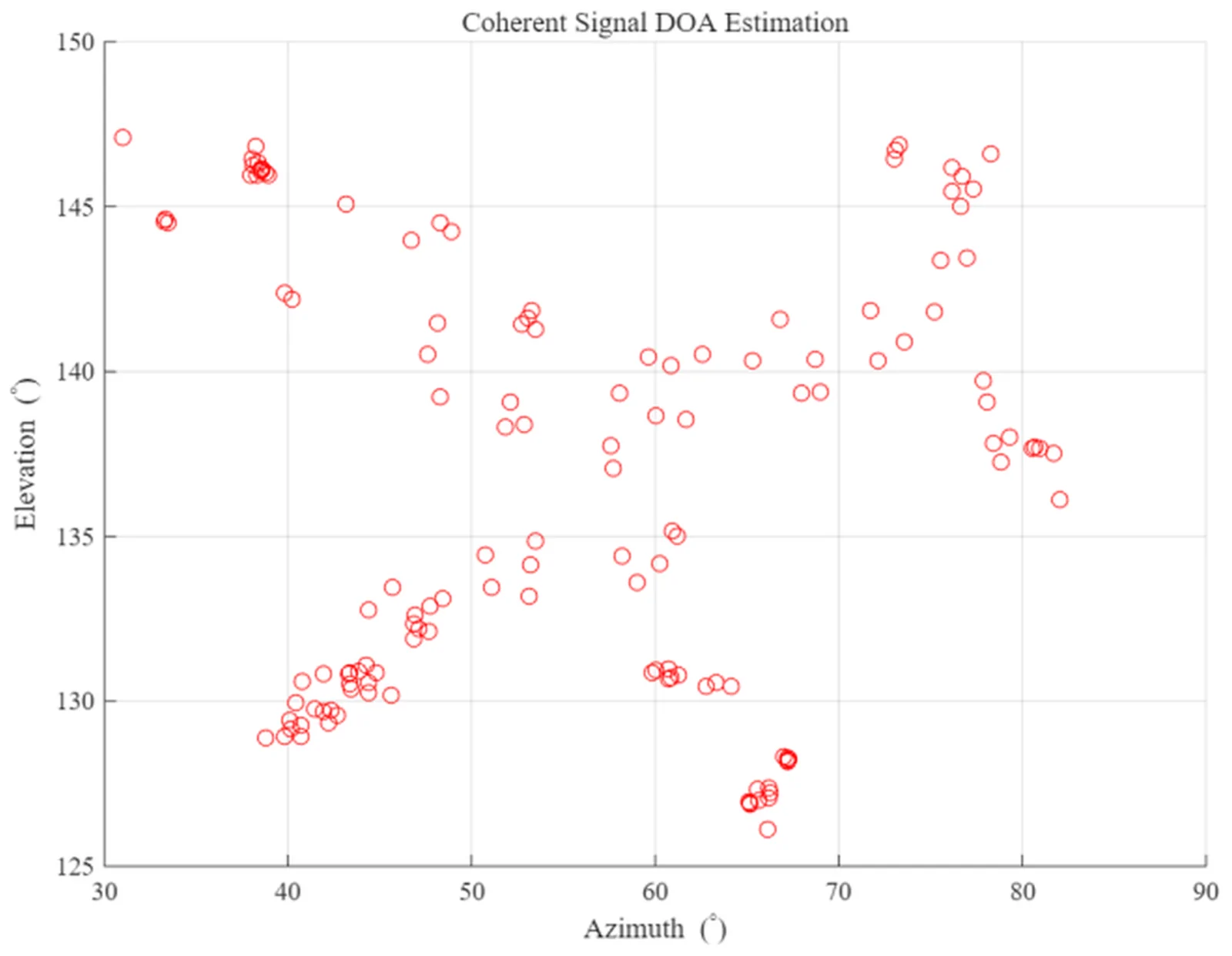
2D DOA joint estimation scatter plot for coherent signals.

Figure 9 showcases the paired 2D coherent DOA estimation results. The scattering centers accurately preserve the macroscopic “cross-shaped” topology of the aircraft’s fuselage and wings. This demonstrates that the proposed cost function pairs angles effectively, suppressing ghost targets while maintaining high spatial resolution.

Finally, by substituting each paired set of true azimuth angle $\phi_{k}$ and elevation angle $\theta_{k}$, along with the target cell’s radial range $R_{d}$ extracted by the CA-CFAR, into the polar-to-Cartesian coordinate transformation matrix, the absolute 3D spatial coordinates of the target are reconstructed.

**Figure 10:**
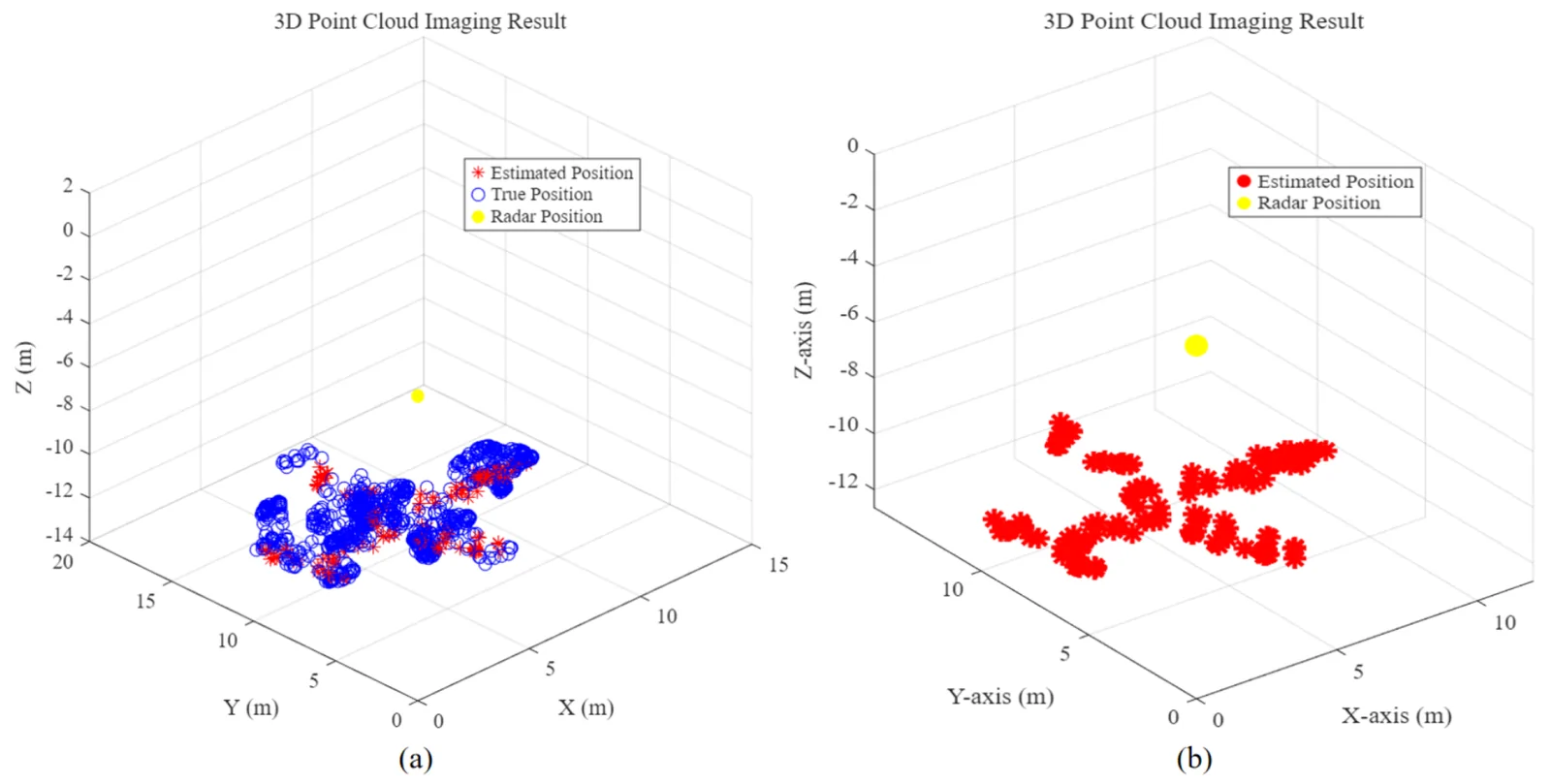
3D point cloud imaging results. (**a**) Real point cloud and estimated point cloud. (**b**) Estimated point cloud.

From Figure 10, it is visually apparent that the reconstructed red point cloud is highly consistent with the contours of the blue ground truth model. Whether it is the longitudinal alignment of the aircraft’s fuselage or the spanwise distribution of the wings on both sides, they are clearly restored in 3D space, with no obvious scale distortion across the Cartesian dimensions (x,y,z). Benefiting from the pairing strategy based on minimizing the Frobenius norm of the cross-covariance matrix, the reconstructed 3D space shows no evident Ghost Targets or discrete outliers caused by mismatches.

### 3.2. Quantitative Evaluation of 3D Point Cloud Reconstruction Quality

**Figure 11:**
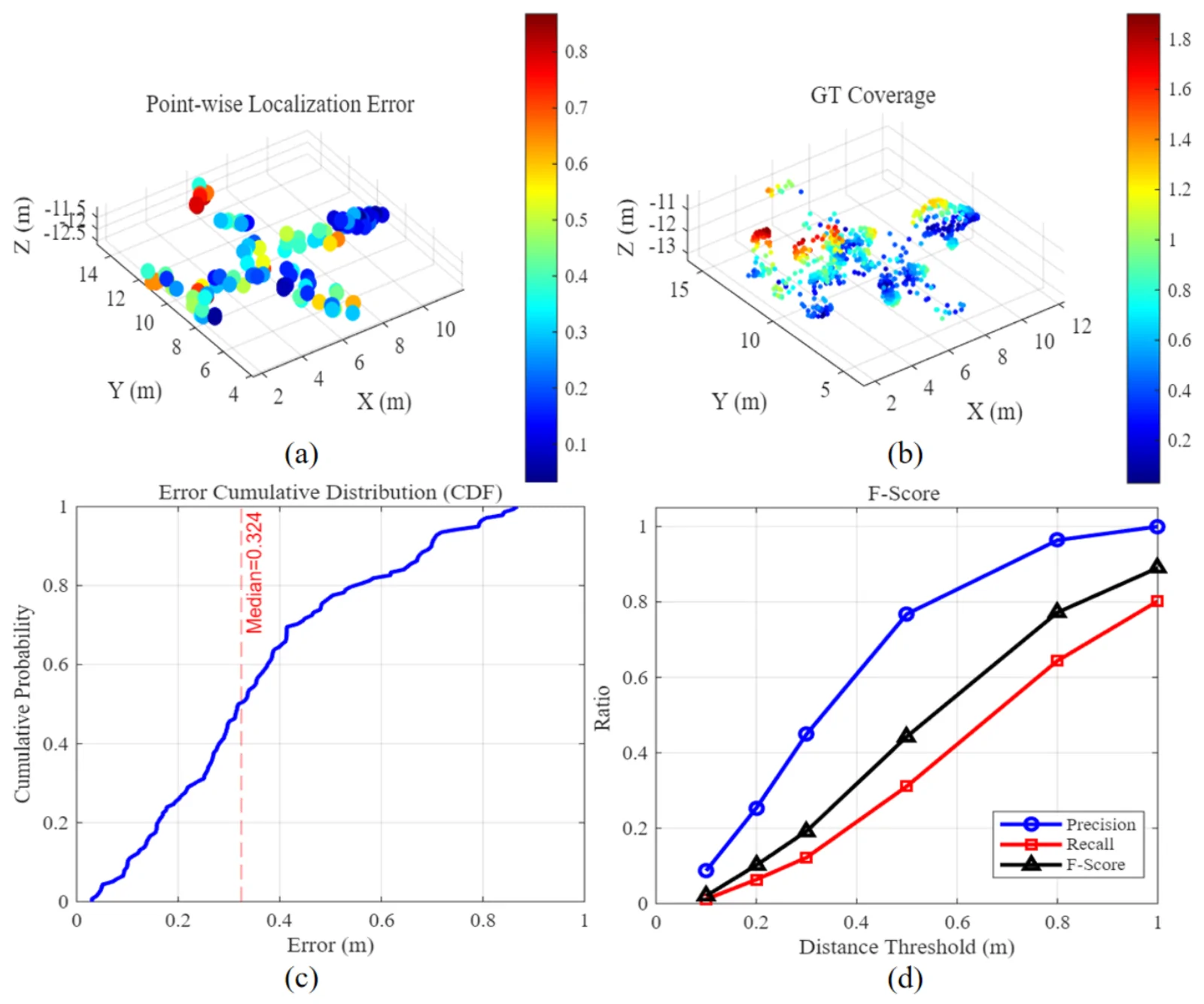
Visual analysis of 3D point cloud imaging quality. (**a**) Point-wise localization error heatmap. (**b**) Ground truth (GT) point coverage heatmap. (**c**) Error CDF curve. (**d**) F-Score versus distance tolerance threshold.

Based on the point cloud evaluation system established in Section 2.4.6, this paper conducted systematic error statistics and quantitative evaluations between the radar-calculated 3D coordinate point cloud set $S_{1}$ and the aircraft’s ground truth spatial physical model set $S_{2}$. Figure 11 illustrates the multidimensional visualization results of the point cloud quality assessment.

The radar system identified $|S_{1}|$ = 138 valid coherent scattering centers from the $|S_{2}|$ = 941 ground truth points. This inherent sparsity reflects the physical mechanism of mmWave radar, which captures discrete strong electromagnetic scattering centers (e.g., nose, wingtips) rather than continuous optical surfaces. To evaluate the 3D reconstruction performance of this algorithm under this physical premise, Table 5 lists the final global error evaluation results of the algorithm across various core 3D evaluation metrics. The Total Chamfer Distance (CD) in the table is computed as the sum of the predicted-to-ground-truth accuracy mean squared error (0.2889 m^2^) and the ground-truth-to-predicted completeness mean squared error (0.6806 m^2^). The bidirectional Hausdorff Distance (HD) is taken as the worst-case value between the maximum unidirectional dislocation from predicted to ground truth (about $h(S_{1},S_{2})=1.01$ m) and from ground truth to predicted (about $h(S_{2},S_{1})=1.80$ m), which is 1.8048 m.

Combining the quantified data in Table 5 with the visual features in Figure 11, the following observations can be drawn:

Figure 11a illustrates the localization error heatmap. Statistical data reveal a mean error of 0.4831 m and a median of 0.3986 m. Coupled with the CDF curve in Figure 11c, approximately 90% of the errors are controlled within 0.8671 m, indicating sub-meter median point-wise localization accuracy in this simulation. Figure 11b displays the ground truth (GT) coverage heatmap. The windward side (nose and leading edges) exhibits dense point cloud coverage with minimal error. Conversely, the leeward side shows sparser coverage, which is consistent with the electromagnetic shadowing effect of the metallic fuselage and the directional characteristics of the target’s RCS.

Angle pairing failures typically generate distant ghost targets. However, the predicted-to-ground-truth Hausdorff component is about $h(S_{1},S_{2})=1.01$ m, indicating that the reconstructed points do not produce large outliers relative to the aircraft contour in this simulation. The larger ground-truth-to-predicted component, about $h(S_{2},S_{1})=1.80$ m, mainly reflects sparse coverage gaps caused by the limited number of radar-visible scattering centers rather than ghost targets. This directional interpretation of HD supports the effectiveness of the Frobenius norm minimization pairing strategy.

To comprehensively evaluate the detection rate, Table 6 lists the F-Score data under various distance tolerance thresholds *d*.

Combining Table 6 and Figure 11d, it is apparent that when the tolerance threshold is set to 1.00 m, the algorithm’s precision reaches 1.00 in this simulation (i.e., 100% of the radar-extracted points are valid true points), and the comprehensive F-Score is 0.8903. Even when the tolerance threshold is tightened to 0.50 m, the algorithm still maintains a high precision of 0.7681.

In summary, both visual and quantitative 3D evaluations demonstrate that the proposed cascaded architecture shows stable point-cloud imaging performance in the considered multi-coherent extended-target simulation scenarios.

## 4. Discussion

### 4.1. Robustness and Boundary Threshold Analysis

To evaluate the performance limits of the proposed algorithm under challenging simulated conditions, Monte Carlo experiments were conducted regarding the background signal-to-noise ratio (SNR) and conformal array curvature.

#### 4.1.1. SNR Threshold Effect Analysis

**Figure 12:**
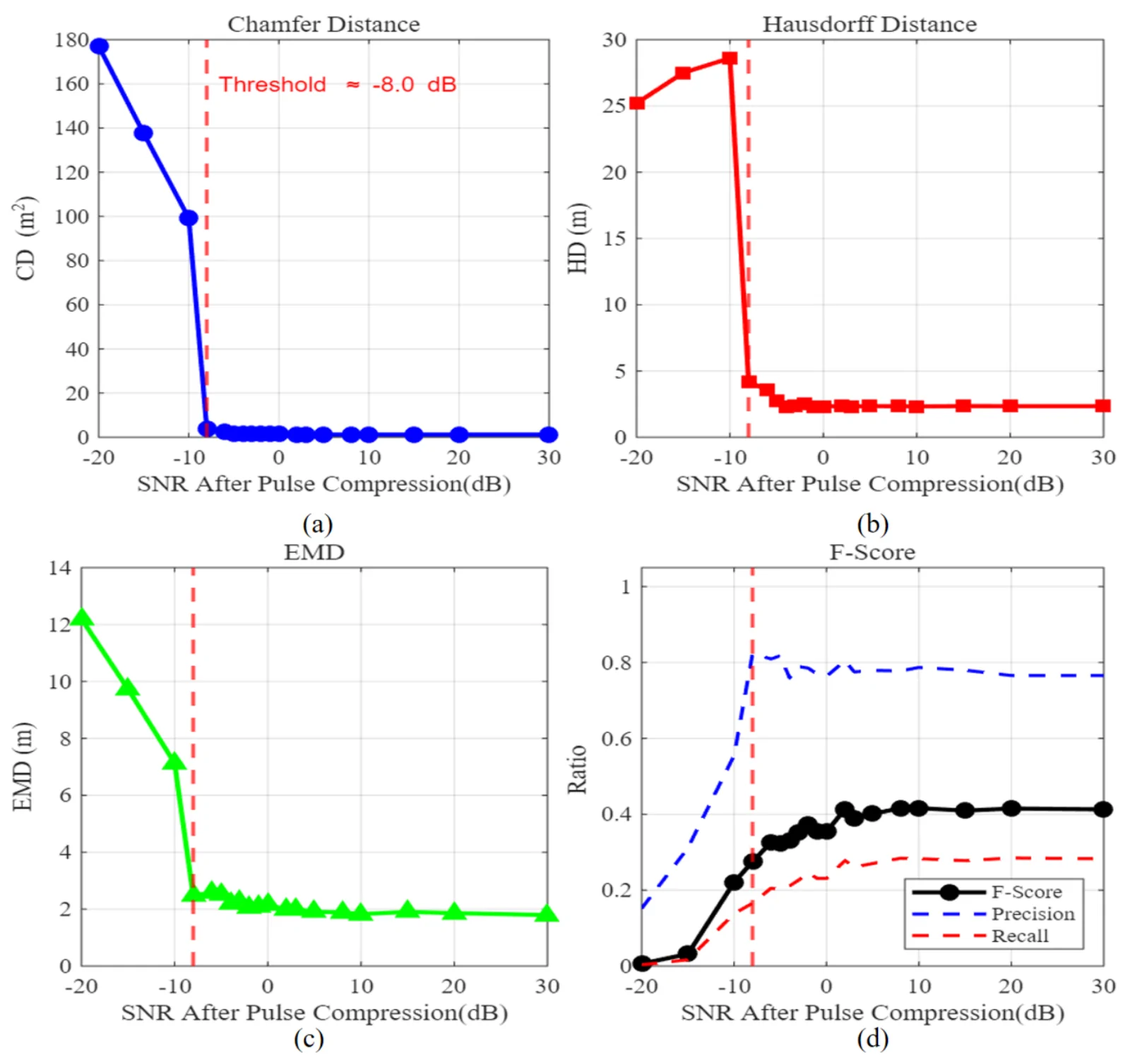
SNR threshold effect and algorithm robustness analysis, where SNR denotes the post-pulse-compression SNR. The red dashed vertical line marks the approximate low-SNR boundary discussed in the text. (**a**) The relationship between CD and SNR. (**b**) The relationship between HD and SNR. (**c**) The relationship between EMD and SNR. (**d**) The relationship between F-Score and SNR at d=0.5 m.

In practical high-speed cylindrical platform applications, radars frequently encounter severe noise interference. To this end, we gradually increased the SNR from −20 dB to 30 dB and systematically compiled the variation trends of the point cloud evaluation metrics and the number of detected targets. In this subsection, SNR denotes the post-pulse-compression SNR after 2D FFT and non-coherent channel integration, and the single F-Score reported in the sweep is computed at the distance threshold d=0.5 m. Table 7 lists the quantitative metrics under several typical SNRs, and Figure 12 presents the complete SNR sweep performance curves.

As shown in Table 7 and Figure 12a–c, within the low-SNR regime (from −20 dB to −10 dB), the system experiences notable reconstruction degradation. At the detection stage, weak scattering-center echoes are partially submerged in the noise floor and many cells fail to exceed the 2D CA-CFAR threshold. Therefore, the number of valid detected points drops to 12 at −20 dB and remains only 32 at −10 dB. The sparse and incomplete detections make the reconstructed point cloud lose the main airframe structure, causing the CD to reach $177.01\;m^{2}$. The HD values are not strictly monotonic in this very low-SNR region: HD is 18.53 m at −20 dB but increases to 28.81 m at −10 dB. This occurs because only 12 points survive detection at −20 dB, so the worst detected-point dislocation is evaluated over a smaller set, whereas more but still unstable detections appear at −10 dB.

The threshold-like transition around SNR ≈ −8.0 dB can be explained by the combined effect of detection recovery and subspace separation. After FBSS, the covariance matrix can be interpreted as a signal-plus-noise matrix, where the useful coherent scattering centers correspond to several dominant eigenvalues and the background noise forms the noise eigenvalue floor. When the SNR is too low, the gap between the weak signal eigenvalues and the noise eigenvalues becomes comparable to the perturbation introduced by AWGN and by the limited number of overlapping smoothed subarrays. In the present single-snapshot implementation, the raw covariance before smoothing is rank-limited, and the usable signal/noise subspace structure is produced by FBSS subarray averaging rather than by many independent temporal snapshots; this interpretation is consistent with classical subspace perturbation theory [42]. In this case, the estimated signal and noise subspaces are poorly separated, the Root-MUSIC polynomial roots deviate from their correct unit-circle locations because of subspace leakage and possible root-swap effects [43], and the subsequent angle pairing may generate large geometric outliers. Once the post-pulse-compression SNR increases to approximately −8.0 dB in this simulation, more scattering centers pass the CA-CFAR detector and the eigenvalue gap becomes sufficiently distinguishable for stable Root-MUSIC rooting. Consequently, the valid detected points rapidly recover to 89, the CD/HD/EMD curves in Figure 12a–c decrease sharply, and the F-Score in Figure 12d rises in a step-like manner to 0.27.

As the SNR further increases toward 0 dB and beyond, both the detection result and the subspace estimate become less sensitive to additional noise reduction, so the point-cloud metrics gradually stabilize. To keep the nominal-condition metrics consistent across the manuscript, the 10 dB row in Table 7 has been updated using the unified r=0.1 m and post-pulse-compression SNR of 10 dB setting: CD, HD, and EMD are 0.97 m^2^, 1.80 m, and 1.95 m, respectively, while the reported F-Score is 0.44 under the d=0.5 m tolerance used in this sweep. Therefore, the −8.0 dB value should be interpreted as a simulation-based transition point determined by the adopted array geometry, FBSS subarray length, CFAR setting, target scattering model, and AWGN assumption, rather than a general physical threshold for all conformal radar systems.

#### 4.1.2. Conformal Curvature Mapping Boundary Analysis

**Figure 13:**
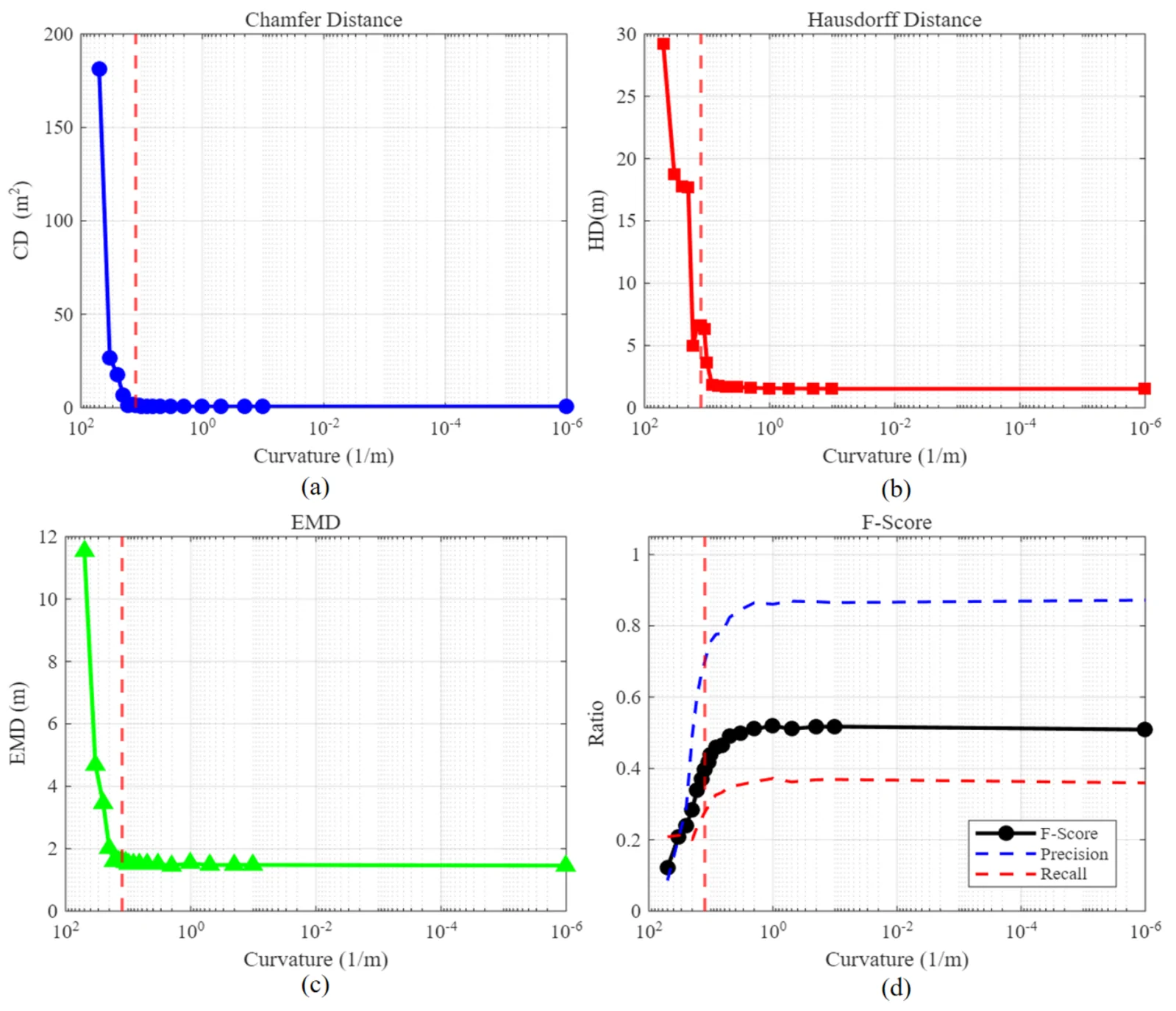
Array curvature mapping error boundary analysis. The red dashed vertical line marks the approximate curvature boundary discussed in the text. (**a**) The relationship between CD and curvature. (**b**) The relationship between HD and curvature. (**c**) The relationship between EMD and curvature. (**d**) The relationship between F-Score and curvature at d=0.5 m.

The core innovation of this paper lies in using matrix T to approximate the non-linear conformal manifold as a ULA manifold. However, strong array bending can exceed the matrix’s linear approximation limit. To investigate this, the array curvature κ=1/r is varied from $50\;m^{-1}$ (strongly curved) to $0\;m^{-1}$ (ideal ULA) to evaluate the impact of mapping errors on 3D imaging quality. Table 8 lists the quantitative metrics under typical curvatures, and Figure 13 displays the complete curvature sweep performance curves.

As detailed in Table 8 and Figure 13, the curvature sweep shows a clear performance transition around $\kappa \approx 12.50\;m^{-1}$ (critical radius r≈0.08 m, indicated by the red dashed line) for the adopted simulation setup. In the moderately curved to flat region ($\kappa \leq 12.50\;m^{-1}$), the theoretical mapping error drops to 0.39 or below. This allows the mapping matrix T to compensate for the non-linear phase differences and approximately maintain the shift-invariance of the equivalent linear array. Consequently, as shown in Figure 13a–c, the geometric errors decrease: CD drops toward $0.65\;m^{2}$, HD decreases from 6.36 m to 1.51 m, and EMD remains in the approximately 1.55–2.05 m range. Concurrently, as depicted in Figure 13d, driven by stable Precision and Recall, the F-Score recovers from 0.42 to around 0.50, approaching the performance of an ideal ULA (κ=0) under the same assumptions. It should also be noted that the nominal operating radius r=0.1 m ($\kappa =10\;m^{-1}$) has only a moderate margin relative to this transition region. Nevertheless, the newly included r=0.1 m row in Table 8 provides a direct cross-check of the nominal operating point and reports CD = 0.97 m^2^, HD = 1.80 m, EMD = 1.95 m, and F-Score = 0.44 under the same post-pulse-compression SNR of 10 dB condition. These values indicate that the degradation around this operating point remains limited compared with the flat-array reference and is much smaller than that in the strongly curved region. Therefore, r=0.1 m is adopted as a practical compromise between conformal installation on a compact cylindrical platform and acceptable manifold-mapping accuracy. For example, at r=0.08 m ($\kappa =12.50\;m^{-1}$), the CD increases to $1.43\;m^{2}$, compared with approximately $0.65\;m^{2}$ in the near-flat/flat reference cases.

Conversely, as the array moves into the strongly curved region ($\kappa >12.50\;m^{-1}$), the mapping error increases substantially (reaching 0.94 at $\kappa =50\;m^{-1}$). This indicates that higher-order non-linear phase errors can exceed the approximation capability of the orthogonal transformation matrix and induce pronounced spatial subspace leakage. As reflected in the table and figures, this leakage causes the maximum dislocation HD to increase to 29.2 m, while CD and EMD increase to $181.2\;m^{2}$ and 11.52 m, respectively. As a result, the F-Score decreases to 0.12, indicating poor 3D point-cloud reconstruction in this simulation case. This curvature sweep therefore provides a simulation-based dimensional reference for conformally embedding mmWave radar arrays into wing leading edges or ogival radomes.

### 4.2. Practical Implementation Considerations and Limitations

The proposed straight-curved L-shaped topology is intended for compact mmWave radar front ends mounted on cylindrical or quasi-cylindrical platform surfaces. Recent conformal-array studies have shown that UAV, airborne, and space-vehicle platforms can employ cylindrical or quasi-cylindrical arrays to improve angular coverage while preserving the aerodynamic envelope, and that microstrip, SIW, slotted-waveguide, and stacked conformal-array implementations are feasible candidates for curved millimeter-wave apertures [9,10,44,45]. In a practical implementation of the proposed topology, the straight arm may be realized using a conventional linear receive array along the longitudinal direction of the carrier. The curved arm can be implemented using flexible or segmented conformal microstrip elements, substrate-integrated-waveguide radiators, slotted waveguide elements, or package-integrated antenna modules bonded to a machined dielectric carrier that follows the local cylindrical surface. For a 77 GHz system, the mechanical tolerance and adhesive-layer thickness should be controlled carefully because small element-position errors become a non-negligible fraction of the wavelength. The curved aperture should also be protected by a low-loss radome or conformal dielectric layer whose thickness and permittivity are included in the measured element pattern and array-manifold calibration.

From the RF front-end perspective, the two arms should share a coherent local oscillator, synchronized chirp generation, and matched receiver chains so that the inter-channel phase relation used by the cross-covariance pairing remains stable. The common reference element should be carefully defined in the mechanical design because it directly affects the cross-covariance used for angle pairing. Practical deployment also requires near-field or anechoic-chamber calibration to measure the embedded element patterns, inter-channel amplitude/phase responses, mutual-coupling matrix, and temperature-dependent phase drift. These measured calibration data can then replace the ideal manifold used in the simulation and can be updated through built-in calibration loops or periodic reference-target measurements.

**Figure 14:**
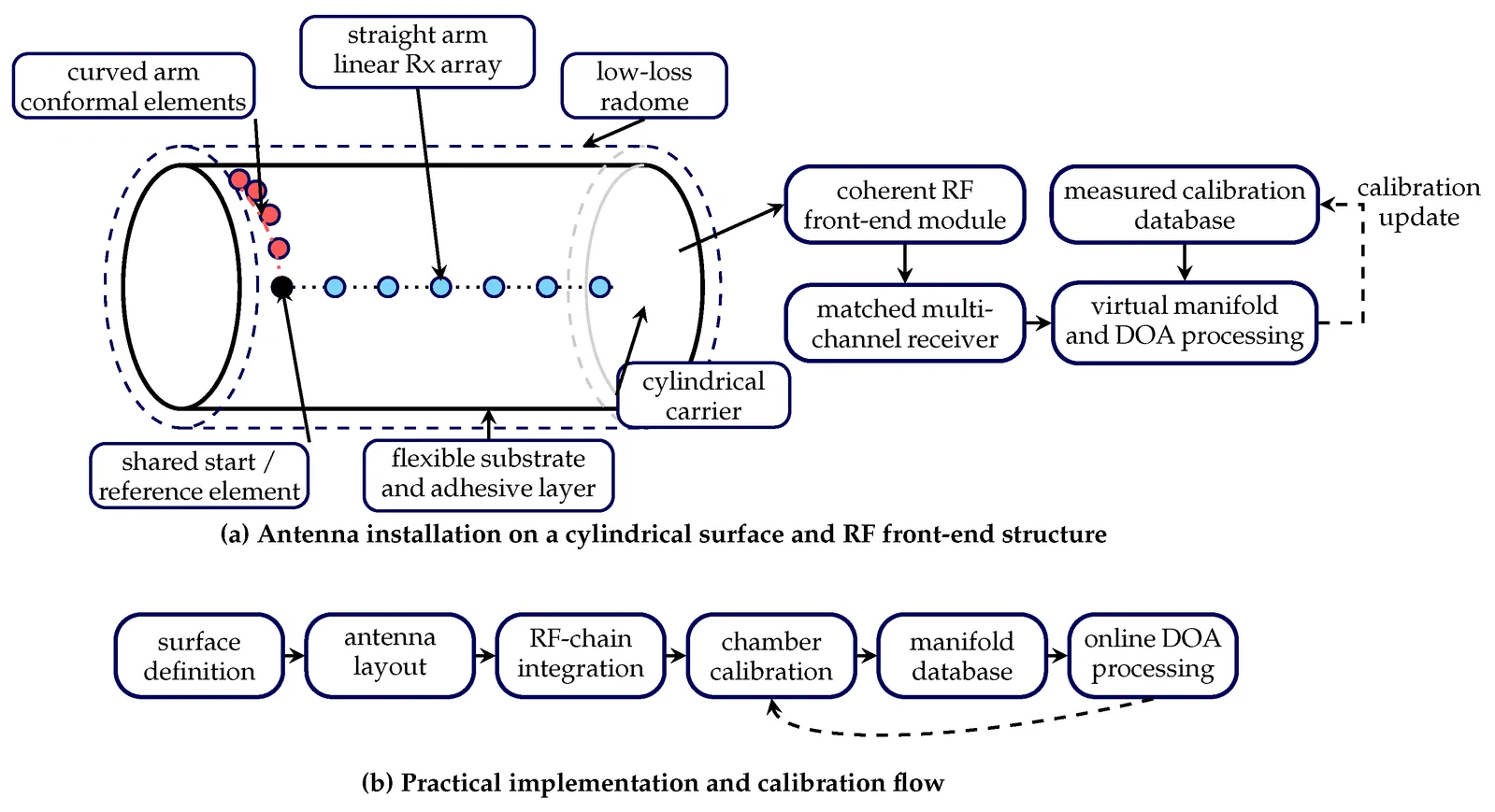
Possible antenna installation, RF front-end structure, and calibration flow for the proposed straight-curved L-shaped conformal array. In panel (**a**), the blue dots denote the straight-arm elements, the red dots denote the curved-arm elements, and the black dot denotes the shared start/reference element.

Figure 14 illustrates a possible implementation route. The straight arm and curved arm are mounted on a shared cylindrical carrier and connected to coherent RF channels. The measured calibration database, including embedded element patterns, channel amplitude/phase responses, mutual coupling, and thermal drift, provides the practical array manifold used by the subsequent virtual transformation and DOA processing stages. As shown in Figure 14a, the implementation can proceed from the mechanical installation to the signal-processing chain. First, a cylindrical carrier is covered by a flexible substrate/adhesive layer and a low-loss radome. The black element is then used as the shared start/reference element, from which the blue elements extend along the cylindrical axis to form the straight linear receive arm and the red elements extend along the local cylindrical contour to form the conformal curved arm. The array ports are connected to a coherent RF front-end module and matched multi-channel receiver so that the inter-channel phase relations are preserved. After chamber or reference-target calibration, the measured embedded element patterns, channel amplitude/phase responses, coupling effects, and thermal drift are stored in the calibration database. This database supplies the practical manifold used by the virtual manifold transformation and DOA processing block. The dashed feedback arrow indicates that the calibration database can be periodically updated and then reused in online processing. The lower part of Figure 14b summarizes this workflow as surface definition, antenna layout, RF-chain integration, chamber calibration, manifold database construction, and online DOA processing.

The present validation is based on MATLAB simulations with prescribed array geometry, AWGN, and a point-scatterer aircraft model. Therefore, the reported SNR threshold, curvature threshold, angular resolution, and point-cloud metrics should be interpreted as simulation-based results under the stated assumptions rather than universal physical limits of all conformal radar systems. Real airborne or spaceborne platforms may introduce additional non-idealities, including element-position errors, gain/phase mismatch, mutual coupling, platform vibration, array deformation, thermal drift, off-grid scattering centers, non-Gaussian clutter, multipath, and target RCS variability. These factors may degrade the manifold transformation accuracy and the eigenstructure used by FBSS/Root-MUSIC.

To provide an initial quantitative robustness check under representative front-end and propagation non-idealities, additional Monte Carlo simulations were conducted using two random seeds for each case. Because two realizations are insufficient for a statistically meaningful standard deviation, the ± values in Table 9 are reported as half-ranges over the two realizations rather than as standard deviations. Table 7 and Table 8 are deterministic single-realization parameter sweeps under the stated nominal seed. The processing algorithm still used the nominal manifold and calibration model, while the received data were generated with residual element-position errors, gain/phase mismatch, mutual coupling, off-grid scattering-center perturbations, or weak multipath. The tested perturbation levels represent calibrated residual errors rather than uncalibrated worst-case hardware faults. High-power impulsive clutter is therefore treated as a harsher clutter scenario for future validation rather than as a mild calibrated residual case in this table. Table 9 summarizes the resulting point-cloud metrics.

As shown in Table 9, mild calibrated non-idealities produce only limited degradation relative to the baseline case. In particular, residual position errors of 0.01λ, gain/phase mismatch of 0.3 dB/3°, mutual coupling with ρ=0.05, 0.05 m off-grid scattering perturbations, and −15 dB multipath all keep the mean point-wise localization error close to the baseline value. Additional stress tests indicated that larger element-position errors (0.02λ and above) and stronger gain/phase mismatch can produce outliers and noticeably increase CD and HD. Therefore, practical deployment should include array geometry calibration, channel amplitude/phase equalization, and mutual-coupling compensation. Future work will further combine measured calibration data, hardware-in-the-loop validation, and electromagnetic co-simulation to quantify robustness under more realistic front-end and propagation conditions.

## 5. Conclusions

This paper proposed a coherent-target 3D point-cloud imaging framework for a straight-curved hybrid L-shaped conformal array on cylindrical airborne platforms. The orthogonal virtual manifold transformation compensates for the conformal phase distortion within a predefined sector, FBSS/Root-MUSIC converts 2D spectral search into reduced-dimensional root finding, and the Frobenius-norm pairing cost reduces angle mismatch and ghost targets.

Under the stated simulation assumptions, the proposed framework recovered the main geometric structure of the aircraft scattering model and achieved a median point-wise localization error of 0.3986 m, CD of 0.9695 m^2^, and EMD of 1.9504 m, with an angular resolution of approximately 1.0° under the specified 10 dB resolution-test condition. The boundary analyses indicated stable reconstruction around a post-pulse-compression SNR of −8.0 dB and a conformal curvature of 12.50 m^−1^ (r=0.08 m). These values are simulation-based design references rather than general physical limits. Future work will validate the method using measured or hardware-in-the-loop data and further examine calibration errors, mutual coupling, platform vibration, clutter, target RCS variability, polarization diversity, and more general propagation models.

## Figures and Tables

**Table 1:** Computational complexity and measured MATLAB runtime of the main processing blocks.

Block	Operation	Complexity	Mean (ms)	Median (ms)
2D range-Doppler FFT	FFT over fast and slow time	$O(MN_{r}N_{d}\log N_{r}N_{d})$	16.39	13.24
Non-coherent integration and CA-CFAR	Power accumulation and sliding-window detection	$O\left(MN_{r}N_{d}\right)+O\left(N_{r}N_{d}\right)$	3.42	3.24
Virtual manifold transformation	Matrix-vector multiplication per detected cell	$O(M_{z}^{2})$	11.31	11.04
FBSS covariance reconstruction	Subarray averaging and covariance formation	$O(K_{s}L^{2})$	2.38	2.26
Root-MUSIC	EVD and polynomial rooting	$O\left(L^{3}\right)+O\left(L^{2}\right)$	7.81	7.63
Angle pairing	Permutation-based Frobenius-norm cost search	$O(K_{s}!K_{s}^{3})$	3.81	3.59
3D point-cloud generation	Spherical-to-Cartesian projection	$O(K_{s})$	0.07	0.01

**Table 2:** Measured MATLAB runtime of representative 2D DOA estimators.

Method	Search Strategy	Mean Runtime (ms)	Median Runtime (ms)
L-shaped 2D-MUSIC	2D spectral search	53.53	51.68
2D Unitary-ESPRIT	Search-free subspace estimation	2.16	1.71
Proposed 2D Root-MUSIC	Polynomial rooting and angle pairing	1.94	1.11

**Table 3:** FMCW radar parameters.

Parameter	Symbol	Value
Carrier frequency	$f_{c}$	77 GHz
Wavelength	λ	3.896 mm
Sampling rate	$f_{s}$	5 MHz
Chirp duration	$T_{c}$	60 μs
Bandwidth	*B*	1.8 GHz
Chirp slope	μ	$B/T_{c}$
Samples per chirp	$N_{sample}$	300
Chirps per frame	$N_{chirp}$	128
Number of frames	$N_{frame}$	1
Coherent sources per detected cell	$K_{s}$	2
FBSS subarray length	*L*	5
Snapshots per detected cell	$N_{\mathrm{snap}}$	1

**Table 4:** Target parameters.

Parameter	Value
Fuselage length	11 m
Wingspan	12.5 m
Aircraft height	3 m
Velocity $\left(v_{x},v_{y},v_{z}\right)$	(20, 0, 0) m/s

**Table 5:** 3D point cloud global error.

Evaluation Metric	Assessment Result
Total Chamfer Distance (CD)	0.9695 m^2^
Bidirectional Hausdorff Distance (HD)	1.8048 m
Earth Mover’s Distance (EMD)	1.9504 m
Point-wise Absolute Localization Error (Median)	0.3986 m

**Table 6:** F-Score versus distance threshold.

Distance Threshold *d* (m)	Precision	Recall	F-Score
0.10	0.0870	0.0117	0.0206
0.20	0.2536	0.0638	0.1019
0.30	0.4493	0.1222	0.1922
0.50	0.7681	0.3114	0.4431
0.80	0.9638	0.6440	0.7721
1.00	1.0000	0.8023	0.8903

**Table 7:** Point cloud quality versus post-pulse-compression SNR; F-Score is computed at d=0.5 m.

SNR (dB)	CD (m^2^)	HD (m)	EMD (m)	F-Score	Valid Detected Points
−20	177.01	18.53	9.92	0.06	12
−10	99.17	28.81	7.84	0.22	32
−8	4.07	4.17	2.91	0.27	89
−5	1.78	2.53	2.34	0.32	92
0	1.40	2.48	2.32	0.35	114
10	0.97	1.80	1.95	0.44	138

**Table 8:** Point cloud quality versus curvature; F-Score is computed at d=0.5 m.

*r* (m)	κ (m^−1^)	Mapping Error	CD (m^2^)	HD (m)	EMD (m)	F-Score
0.02	50.00	0.94	181.2	29.2	11.52	0.12
0.05	20.00	0.60	6.73	17.7	2.03	0.28
0.08	12.50	0.39	1.43	6.36	2.05	0.42
0.10	10.00	0.30	0.97	1.80	1.95	0.44
0.15	6.67	0.21	0.76	1.76	1.70	0.48
1.00	1.00	0.03	0.66	1.56	1.60	0.51
∞	0.00	0.00	0.65	1.51	1.55	0.50

**Table 9:** Non-ideal robustness simulation under calibrated residual errors at a post-pulse-compression SNR of 10 dB. The ± values denote half-ranges over two realizations rather than standard deviations.

Case	Non-Ideal Condition	CD (m^2^)	HD (m)	Mean Error (m)
Baseline	Ideal array + AWGN	0.97±0.01	1.80±0.01	0.48±0.00
Position error	$\sigma_{p}=0.01\lambda$	1.05±0.12	1.85±0.12	0.53±0.08
Gain/phase residual	0.3 dB/3°	1.00±0.09	1.84±0.05	0.50±0.05
Mutual coupling	ρ=0.05	0.97±0.00	1.80±0.03	0.48±0.00
Off-grid scatterers	$\sigma_{s}=0.05$ m	1.39±0.19	2.19±0.50	0.50±0.03
Multipath	−15 dB weak echo	1.05±0.07	1.86±0.06	0.49±0.00

## Data Availability

The original contributions presented in this study are included in the article. Further inquiries can be directed to the corresponding author.

## Abbreviations

The following abbreviations are used in this manuscript:

DOA	Direction of arrival
FMCW	Frequency-modulated continuous-wave
FBSS	Forward-Backward Spatial Smoothing
ULA	Uniform linear array
RDM	Range-Doppler Map
CFAR	Constant false alarm rate
CD	Chamfer Distance
HD	Hausdorff Distance
EMD	Earth Mover’s Distance
